# Structures and molecular mechanisms of RAD54B in modulating homologous recombination

**DOI:** 10.1038/s41467-026-78608-4

**Published:** 2026-10-12

**Authors:** Pengtao Liang, Stephanie Tye, Johanna Ertl da Costa, Neelam Maharshi, Bilge Argunhan, Lucas Kuhlen, Megan Battley, Elizabeth A. McCormack, Wolf-Dietrich Heyer, Markus Löbrich, Xiaodong Zhang

**Affiliations:** 1 Section of Structural and Synthetic Biology, Faculty of Medicine, Imperial College London, London, UK; 2 Laboratory of DNA Processing Machines, Francis Crick Institute, London, UK; 3 Radiation Biology and DNA Repair, Technical University of Darmstadt, Darmstadt, Germany; 4 Department of Microbiology and Molecular Genetics, University of California, Davis, Davis, CA USA

**Keywords:** Homologous recombination, Cryoelectron microscopy, Double-strand DNA breaks

## Abstract

Genome stability is essential for cellular viability yet constantly threatened by DNA-damaging agents. Among these, DNA double-strand breaks (DSBs) are particularly harmful and in S/G2 phases are faithfully repaired through homologous recombination (HR). The RAD51 recombinase forms nucleoprotein filaments on single-stranded DNA (ssDNA) that performs critical functions in HR. The efficiency of HR depends on precise regulation of RAD51 filament dynamics by accessory factors, including RAD54B, a double-strand DNA-dependent ATPase. Here, using cryogenic electron microscopy, mutagenesis, and biochemical assays, we show that RAD54B stabilizes RAD51–DNA filaments, inhibits RAD51 ATPase activity, and promotes D-loop formation and strand exchange. RAD54B contains two main RAD51-interacting sites and a β-domain that bridges RAD51 protomers, is positioned to contact donor dsDNA, and regulates RAD54B’s ATPase activity. Cellular assays reveal that the RAD51-interacting sites are required for repairing DSBs by HR in human cells. Our findings uncover a modular architecture and mechanistic framework for RAD54B’s critical role in genome maintenance.

## Abstract (web summary)

RAD54B contains two main RAD51-interacting sites and a β domain that bridges RAD51 protomers, is positioned to contact donor dsDNA, and regulates RAD54B’s ATPase activity. Cellular assays reveal that the RAD51-interacting sites are required for repairing DSBs by HR in human cells

## Introduction

Maintaining genome stability is fundamental to cellular functions. However, cells are constantly challenged by exogenous agents such as chemical mutagens and ionizing radiation, and endogenous cellular metabolites that can induce a variety of DNA lesions^1^. Among these, DNA double-strand breaks (DSBs) represent one of the most deleterious forms of damage, as they can directly lead to the loss of essential genetic information. Homologous recombination (HR) is a key pathway that repairs DSBs with high fidelity and also plays a critical role in stabilizing stalled or collapsed replication forks^2,3^. Dysregulated HR can compromise genome integrity and drive tumorigenesis^4,5^.

HR is initiated when nucleolytic resection generates single-stranded DNA (ssDNA) overhangs at the DSB site. These ssDNA ends are rapidly coated by replication protein A (RPA) and subsequently displaced by the RecA-family recombinase RAD51, or its meiosis-specific counterpart DMC1, which form helical nucleoprotein assemblies known as presynaptic filaments. These filaments perform the essential task of homology search by interrogating double-stranded DNA (dsDNA) for sequence complementarity. Upon locating a homologous sequence, the filament mediates strand invasion, pairing the ssDNA with its complementary strand in the dsDNA to form a displacement loop (D-loop), which then leads to DNA synthesis and subsequent repair^2,6^. RAD51 filaments are therefore central components of the HR pathway.

The efficiency and accuracy of HR rely on precise regulation of RAD51–DNA filament assembly, dynamics, and disassembly by a range of accessory factors^7^. Furthermore, the intrinsic recombinase activity of RAD51 is low and requires modulators to stimulate. Among these modulators, the SWI2/SNF2-family dsDNA translocases RAD54 and RAD54B are ATP-driven motor proteins that directly bind RAD51 or DMC1 filaments and stimulate recombination activities^8–13^. RAD54 has been well characterized as a dsDNA-dependent ATPase that plays important roles in HR by stimulating RAD51 recombinase activities^14^. Both yeast and human RAD54 were shown to bind to RAD51 and dissociate RAD51 from double-stranded DNA; therefore, a role for disassembling RAD51 from heteroduplex DNA in D-loops has been proposed^15–19^. The exact roles of RAD54B in homologous recombination are less well understood. Yeast ortholog Rdh54 (also known as Tid1) was shown to play a role in both mitotic and meiotic recombination^20,21^. In mitotic recombination, Rdh54 was proposed to antagonize Rad54 during Rad51-mediated recombination^22^ and acts as an overall negative regulator of D-loop formation^23^. Very recently, human RAD54 and RAD54B were shown to function in two distinct sub-pathways in homologous recombination, with RAD54 in double-Holliday junction resolution, while RAD54B in synthesis-dependent strand annealing^24^.

In vitro, Rdh54 binds directly to Rad51 filaments and uses ATP hydrolysis to translocate along DNA, remodel recombinase–DNA complexes, and in some contexts, displace Rad51^25^. Human RAD54B was also shown to directly interact with both RAD51 and DMC1 and can stimulate recombinase-driven strand invasion or strand exchange in vitro^26,27^.

To understand how human RAD54B modulates RAD51 recombination activities and which aspects it modulates, and to characterize RAD54B in DSB repair, we set out to characterize the molecular mechanisms and functions of RAD54B in HR. Using cryo-electron microscopy (cryo-EM) combined with targeted mutagenesis, biochemical reconstitution, and cellular assays, we define the molecular basis by which RAD54B engages and modulates RAD51 filaments to regulate HR. Our results show that RAD54B inhibits the ATPase activity of DNA-bound RAD51, stabilizes recombinase filaments, and promotes strand invasion, D-loop formation, and strand exchange, thereby facilitating efficient HR. Interestingly, RAD54B does not dissociate RAD51 from dsDNA.

Specifically, we show that the RAD54B-NTD is sufficient to stabilize RAD51 filaments and promote strand exchange, but not D-loop formation, which requires the C-terminal ATPase domain. This domain-specific functionality suggests a modular architecture in which the NTD mediates key protein-protein and protein-DNA interactions, while the ATPase domain provides the motor activity necessary for DNA remodeling during strand invasion. Structural and mutational analyses further reveal that the NTD contains three distinct RAD51-interacting sites, which are essential for filament stabilization. Importantly, the third binding site (β-domain) that bridges two distal RAD51 protomers in the filament can also interact with dsDNA via a positively charged loop and promote strand invasion and D-loop formation. Further, we show that the β-domain regulates RAD54B DNA-dependent ATPase activity and promotes functional higher-order organization, suggesting a regulatory role of NTD on CTD. We also assess DSB repair by HR in human cells and show that RAD54B knockout (KO) cells exhibit a defect in repairing camptothecin-induced DSBs. The repair defect is rescued after transfection with wild-type RAD54B but not with RAD54B containing deletions in the NTD RAD51 interaction sites or mutations in the β-domain. Together, we demonstrate that the three sites have distinct functions in regulating RAD51 and HR activities, providing a structural framework for understanding their role in HR.

## Results

### RAD54B enhances RAD51 recombination activities via direct interactions

**Fig. 1:**
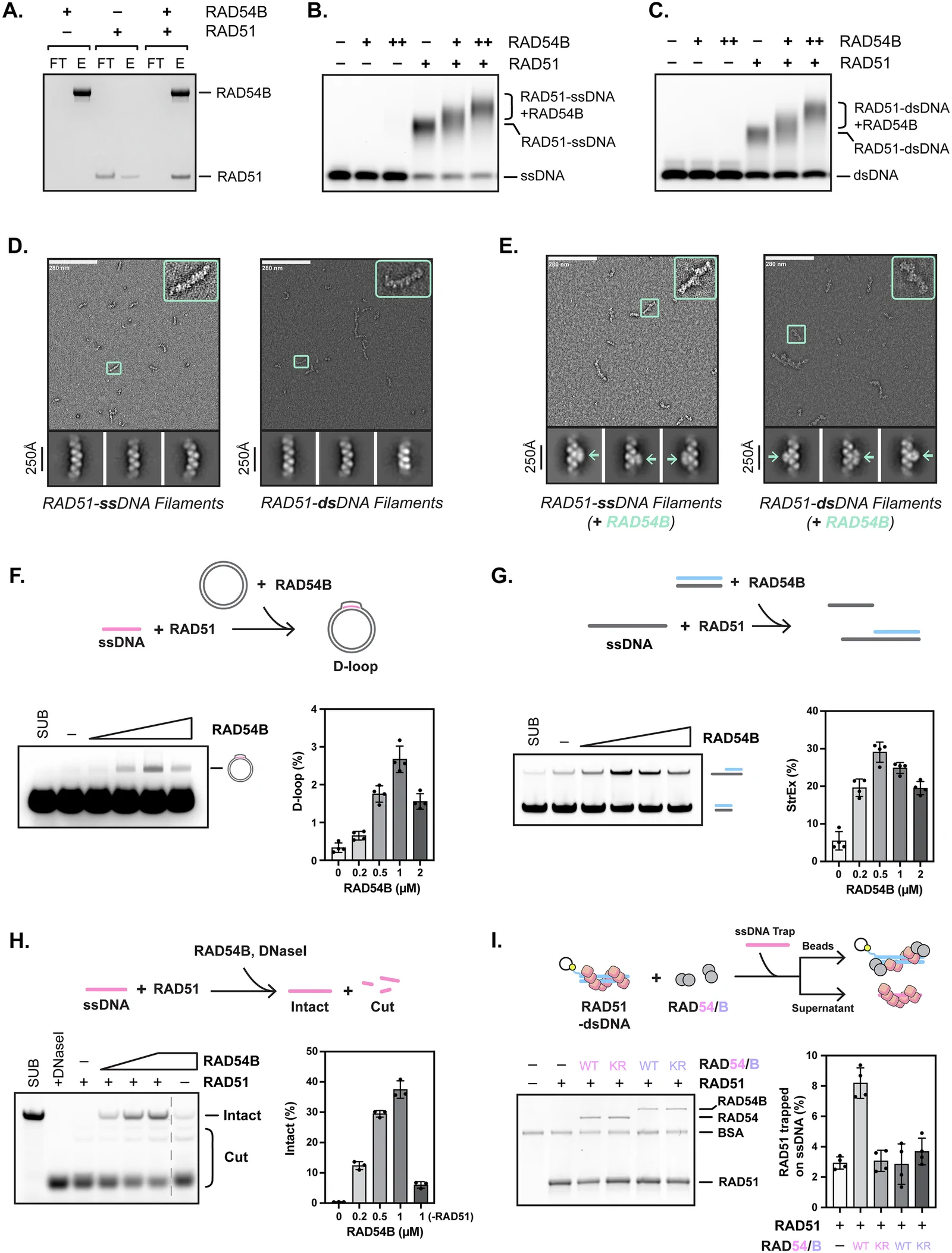
RAD54B stimulates RAD51-mediated recombination by binding to its filament form. **A** Pull-down experiment of full-length RAD54B with RAD51, 1 µM protein for each. Elution (E) and flow-through (FT). **B** Electrophoresis mobility shift assay (EMSA) showing RAD54B (0.5 and 1 µM) binding to RAD51–ssDNA filaments. **C** Electrophoresis mobility shift assay (EMSA) showing RAD54B (0.5 and 1 µM) binding to RAD51–dsDNA filaments. **D** Negative-stain electron microscope micrographs and 2D class averages of RAD51 -ssDNA or -dsDNA filaments. **E** Negative-stain electron microscope micrographs and 2D class averages of RAD51 -ssDNA or dsDNA filaments in the presence of full-length RAD54B. **F** D-loop assay. Schematic (top), representative gels (left), quantification (right) showing stimulation of RAD51-driven strand invasion (1 µM, 6 µM nt, 60 µMbp) by full-length RAD54B (0.2, 0.5, 1, and 2 µM). **G** Oligonucleotide strand exchange assay. Schematic (top), representative gels (left), quantification (right) showing stimulation of RAD51-driven strand exchange (1 µM, 3 µM nt, 1 µMbp) by full-length RAD54B (0.2, 0.5, 1, and 2 µM). **H** ssDNA Nuclease protection assay. Schematic (top), representative gels (left), quantification (right) showing RAD51–ssDNA (1 µM, 10 µM nt) filaments stabilization by full-length RAD54B (0.2, 0.5, and 1 µM). **I** RAD51 displacement assay. SDS-PAGE followed by SYPRO-Orange staining of eluted fractions from magnetic beads containing dsDNA-bound proteins. Graphs comparing the percentage of RAD51 displaced by RAD54 and RAD54B. The representative experiments shown in (**A**) and (**B**) were independently repeated three times with similar results. The experiment shown in (**C**) was independently repeated twice with similar results. Representative EM images and corresponding 2D class averages shown in (**D**, **E**) were generated from four separate datasets corresponding to four samples shown. Each dataset comprises approximately 200 micrographs. For (**F**–**I**), data are presented as mean ± SD from *n* independent biochemical experiments (*n* = 4 for **F**, **G**, and **I**; *n* = 3 for **H**).

Previous biochemical studies have shown that human RAD54B interacts with RAD51^28^. More recently, single-molecule analysis demonstrated that the yeast ortholog Rdh54 binds directly to RAD51–ssDNA filaments^29^. To investigate how RAD54B engages with RAD51 and modulates its activities, we carried out in vitro studies using purified proteins (Fig. 1 and Supplementary Fig. 1A). We first assessed the interactions between RAD54B and RAD51, as well as their respective DNA filaments. As expected, RAD51 can be pulled down by RAD54B (Fig. 1A), and these interactions extended to both ssDNA and dsDNA filament forms, as evidenced by the altered migration patterns of the filaments during electrophoresis (Fig. 1B, C), indicative of complex formation. To visualize these complexes, we employed negative-stain electron microscopy. 2D class averages of images of RAD51–ssDNA or -dsDNA complexes alone showed regular helical filaments (Fig. 1D). Upon addition of RAD54B, both types of filaments were decorated with additional density on the side of the filaments (Fig. 1E). Closer inspection of the 2D class averages revealed two-lobed densities (Fig. 1E, arrows), consistent with binding by RAD54B, which is predicted to have a bi-lobed structure consisting of two RecA-like domains. Interestingly, contrary to previous studies suggesting that RAD54B preferentially associates with filament ends^26^, our data suggest that RAD54B can bind along the length of RAD51 filaments.

The yeast Rdh54 was shown to stimulate RAD51-driven strand invasion^30^. To investigate if human RAD54B has similar effects, we used a plasmid DNA-based D-loop assay^31^. RAD51 was pre-incubated with a 90-nt fluorophore-labeled ssDNA designed to target a unique homologous sequence in the plasmid DNA. Upon successful strand invasion, the RAD51–ssDNA filament displaces the non-complementary strand of the plasmid, forming a D-loop. The fluorophore label allowed detection of stable D-loop products by electrophoresis. Under the conditions employed here, RAD51 alone produced no detectable stable D-loops (Fig. 1F). The addition of RAD54B enhanced D-loop formation in a concentration-dependent manner with peak enhancement at ~1 µM.

To assess strand exchange activities upon strand invasion, we employed an oligonucleotide strand exchange assay. Here, RAD51–ssDNA filaments were first formed on 116-nt single-stranded DNA, and then incubated with homologous, fluorescently labeled 60-bp double-stranded DNA (dsDNA). During the reaction, the ssDNA pairs with the fluorescently labeled complementary strand, displacing the non-complementary strand and generating a heteroduplex composed of 60-bp dsDNA and a 56-nt ssDNA overhang (Fig. 1G). Substrates and products can be resolved by electrophoresis due to their distinct sizes, as visualized via the fluorescent label. Consistent with the strand invasion results, RAD54B enhanced the RAD51-driven strand exchange in a concentration-dependent manner that peaks around 0.5–1 µM (Fig. 1G).

The stimulatory effects could be due to enhanced filament stabilization as proposed for other RAD51 modulators such as RAD51AP1 and RAD51 paralogs^32–34^. We next investigated its effects on RAD51 filaments' stability using nuclease protection assays. The results showed an increased stabilization with increasing RAD54B concentrations (Fig. 1H and Supplementary Fig. 1B).

Unlike human RAD54 and yeast Rad54 and Rdh54, which have been shown to displace RAD51 from dsDNA using its ATPase activities^25,35,36^, RAD54B does not remove RAD51 from dsDNA under these experimental conditions (Fig. 1I). Indeed, using nuclease protection assays, we observe enhanced stability of RAD51–dsDNA filaments (Supplementary Fig. 1B).

### N-terminal domain of RAD54B is sufficient to modulate RAD51 filaments

**Fig. 2:**
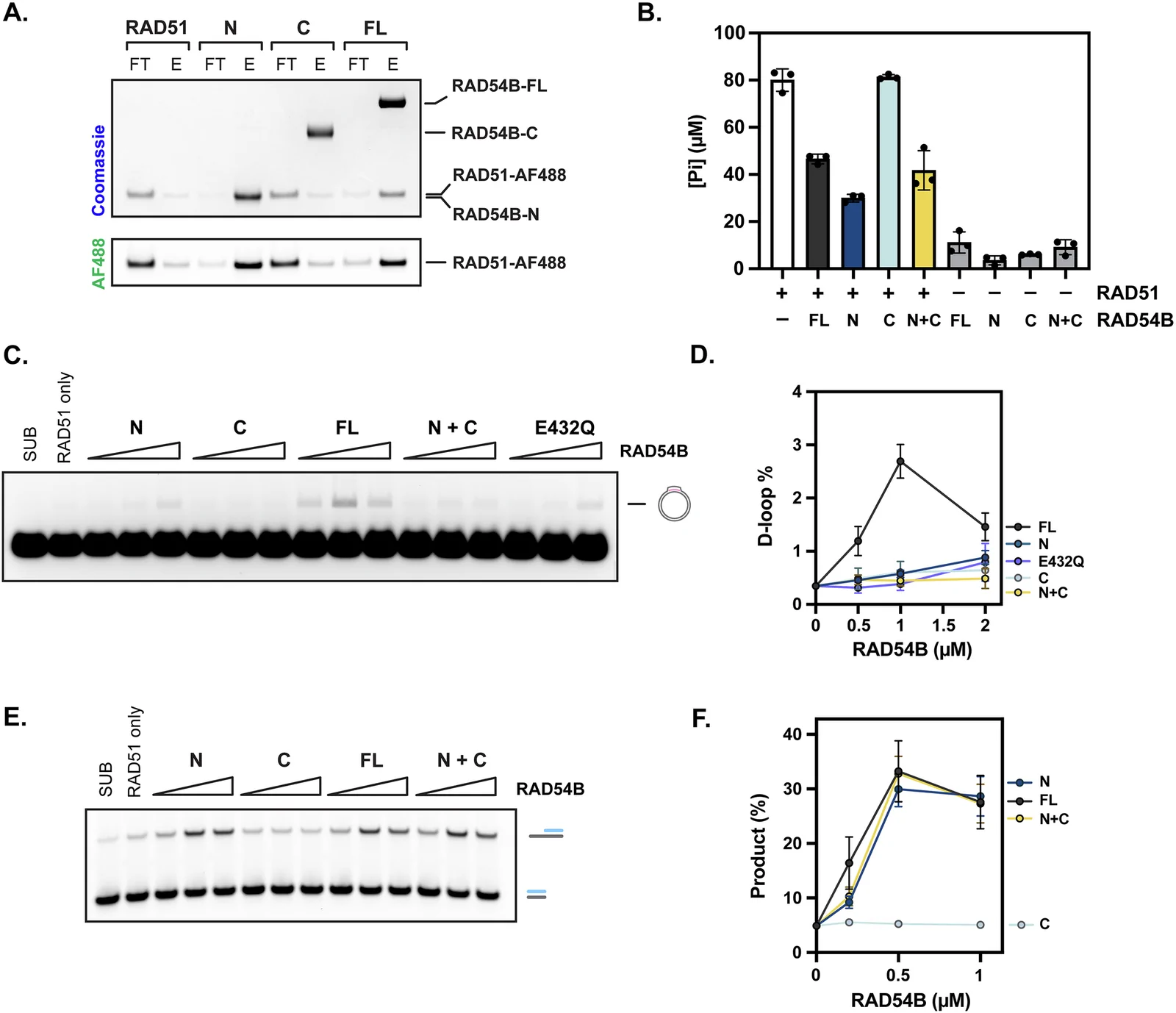
The N-terminal region of RAD54B is sufficient to recapitulate most activities in regulating RAD51. **A** Pull-down assay of RAD54B fragments (N-terminal, C-terminal, and full-length [FL]) with RAD51. The top gel was imaged after staining with Coomassie blue, and the bottom gel was imaged with Aurora Flour 488. **B** ATPase assay showing inhibition of RAD51 ATPase activity in ssDNA filaments by the N-terminal of RAD54B. **C** Representative gel images showing the stimulation of RAD51-driven D-loop formation by RAD54B (full-length, fragments, and mutant) (0.5, 1, and 2 µM). **D** Quantification of (**C**). **E** Representative gel images showing the stimulation of RAD51-driven strand exchange by RAD54B (N, C, and FL) (0.2, 0.5, and 1 µM). **F** Quantification of (**E**). For (**B**–**F**), data are presented as mean ± SD from *n* independent biochemical experiments (*n* = 3).

RAD54B contains a largely disordered N-terminal domain and a conserved C-terminal ATPase domain (Supplementary Fig. 1C, D)^13^. Previous studies identified the NTD of human RAD54B to be responsible for mediating its interaction with RAD51^28^. To assess their functions in RAD51 recombination activities, we purified the NTD (residues 1–285) and the CTD (residues 286–910) separately. Consistent with previous reports, the NTD (RAD54B-N) efficiently pulled down RAD51, similarly to full-length proteins (Fig. 2A). By contrast, CTD (RAD54B-C) showed only minimal interactions, similarly to the background signal.

Since some RAD51-interacting proteins regulate RAD51 ATPase activity, we examined whether RAD54B, and in particular its N-terminal domain, modulates RAD51 ATPase function. RAD54B alone has negligible ATPase activity under these experimental conditions (Fig. 2B), consistent with the observation that it is a dsDNA-dependent ATPase^10^. Both full-length RAD54B and RAD54B-N inhibited RAD51 ATPase activity when RAD51 was assembled into ssDNA filaments, whereas the C-terminal domain of RAD54B had no effect (Fig. 2B), consistent with its inability to bind RAD51 (Fig. 2A).

Since RAD54B-N recapitulates the binding and ATPase-modulating activity of the full-length protein, we asked whether it could also modulate RAD51-driven D-loop formation and strand exchange. In D-loop assays, both RAD54B-N and RAD54B-C failed to substitute for full-length RAD54B (Fig. 2C, D). Notably, in oligonucleotide strand exchange assays, RAD54B-N recapitulated the activity of the full-length protein, whereas the C-terminal domain of RAD54B had no detectable effect (Fig. 2E, F). These results suggest that, while the NTD of RAD54B is sufficient to promote strand exchange, the C-terminal ATPase domain is indispensable for promoting a stable D-loop formation. This notion is further supported by analysis of a RAD54B Walker B mutant (E432Q) that abolishes ATP hydrolysis. While this mutant retained the ability to stimulate strand exchange (Supplementary Fig. 1E), it was defective in promoting D-loop formation (Fig. 2C, D), consistent with previous findings for RAD54, which also requires an intact ATPase site for its recombination activities^37^ and highlighting the differences in these two assays in probing recombinase activities.

Taken together, our data demonstrate that RAD54B-N is essential and sufficient to mediate binding to RAD51, inhibit RAD51 ATPase activity, and modulate strand exchange activities. However, full-length RAD54B, including an intact ATPase site, is required to support the D-loop formation.

### Structural basis of RAD54B interaction with RAD51–DNA filaments

To provide a structural and molecular basis for how RAD54B engages and modulates RAD51–DNA filaments, we employed cryo-electron microscopy (cryo-EM) single particle analysis to obtain the structures of RAD54B-N in complex with RAD51–DNA filaments.

**Fig. 3:**
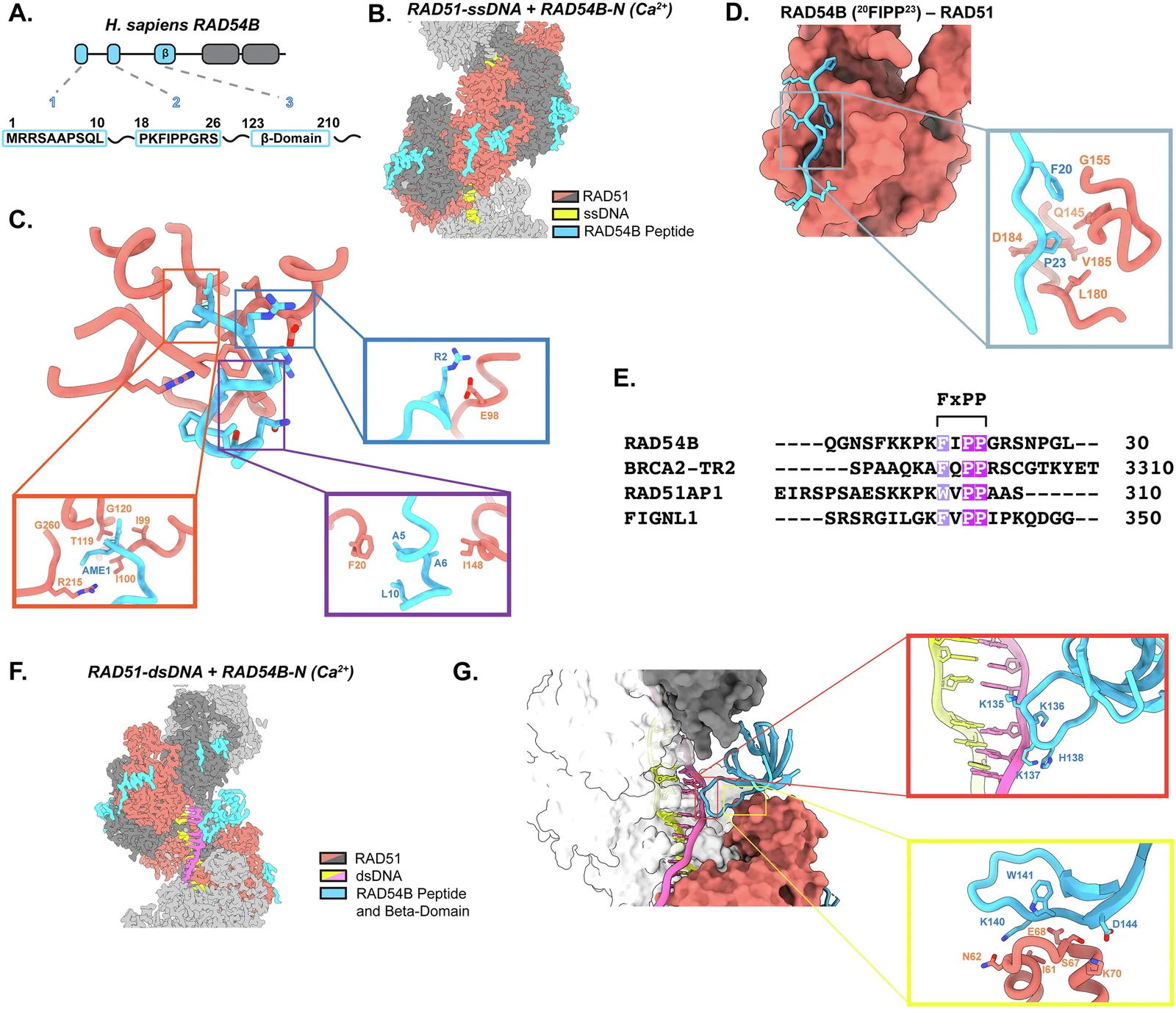
Cryo-EM structure of the RAD51–DNA filaments in complex with the N-terminal domain of RAD54B. **A** Schematic of three distinct RAD51-binding sites in RAD54B-N. **B** Cryo-EM map of the Ca^2+^-ATP-bound RAD51–ssDNA filament in the presence of RAD54B-N. **C** Close-up view of the RAD54B site 1 (blue) interacting with a RAD51 protomer (salmon). **D** Interaction between RAD54B site 2 (^20^FIPP^23^) (blue) and a RAD51 protomer (salmon). **E** Sequence alignment of RAD51 regulators, highlighting the conserved FxPP motif. **F** Cryo-EM map of the Ca^2+^-ATP-bound RAD51–dsDNA filament in the presence of RAD54B-N. **G** Structural details of the RAD54B β-domain interacting with RAD51–dsDNA filament.

We reconstituted RAD51–ssDNA filaments in complex with RAD54B-N for cryo-EM analysis. Image processing produced 3D reconstructions of RAD51–ssDNA–RAD54B-N complexes at a global resolution of 2.6 Å in the presence of ATP hydrolysis-inhibited Ca^2+^-ATP (Fig. 3 and Supplementary Fig. 2A) and 2.9 Å in the presence of Mg^2+^-ATP (Supplementary Fig. 2B, 1st Workflow Path, Supplementary Fig. 3A, Table 1). The two reconstructions are similar and have sufficient resolution to build a structural model. Both reconstructions revealed densities corresponding to binding sites 1 and 2 of the RAD54B-N (Fig. 3A, Supplementary Fig. 3B), contacting each RAD51 protomer (Fig. 3B). Site 1 involves the extreme N-terminal 10 residues of RAD54B, including the Met1, which is acetylated in our proteins as confirmed by mass spectrometry. There are extensive and specific interactions: the acetylated methionine (AME1) inserts into a hydrophobic pocket on RAD51 while Arg2 forms a salt bridge with RAD51 residue Glu98. Additional hydrophobic interactions are mediated by Ala5, Ala6, and Leu10 from RAD54B and Phe20 and Ile148 from RAD51 (Fig. 3C). Site 2 includes an FxPP motif (^20^FIPP^23^) that is conserved in several RAD51 modulators including BRCA2, FIGNL1 and RAD51AP1 (Fig. 3A, D, E)^38^, where Phe20 and Pro23 from RAD54B interact with a hydrophobic pocket on RAD51 filament surface (Fig. 3D).

**Table 1:** Cryo-EM data collection and model statistics

	RAD51 + RAD54B-N				RAD51 + RAD54-N
Nucleotide	ATP-Ca^2+^	ATP-Mg2+	ATP-Mg2+	ATP-Ca2+	ATP-Ca2+
DNA substrate	ssDNA	ssDNA	ssDNA	dsDNA	ssDNA
Microscope	Titan Krios				Glacios
Voltage (keV)	300				200
Detector	K3 (Gatan)				Falcon 4
Magnification	105,000				100,000
Defocus range	−0.6 to −2				−0.4 to −2
Frames/movie	60				
Pixel size	0.84				1.14
Electron dose	60				
Exposure (s)	3.2				
Movies	6086	25,595	25,595	14,860	8876
Picked particles	5,171,621	1,690,118	1,690,118	2,317,245	1,754,084
Final particles	755,548	1,258,150	148,663	298,461	1,388,876
Processing method	Single particle analysis	Single particle analysis	Single particle analysis	Single particle analysis	Single particle analysis
Resolution	2.6	2.9	3	2.4	3.2
Real-space refinement	Non-uniform refinement	Homogeneous refinement	Non-uniform refinement	Homogeneous refinement	Helical refinement
***Composition***					
Non-H atoms	15,809	15,853	16,210	16,602	15,361
Protein residues	1986	1986	2031	2047	1926
Nucleotide residues	18	20	20	35	18
Ligands	12	17	17	18	18
Cross correlation (CC, mask)	0.9	0.87	0.85	0.91	0.89
***Bonds (rmsd)***					
Lengths (Å)	0.002	0.003	0.004	0.003	0.003
Angles (°)	0.464	0.532	0.992	0.732	0.525
MolProbity score	1.07	1.36	1.56	1.25	1.49
Clash score	2.64	6.13	8.13	4.8	9.28
Rotamer outliers (%)	1.07%	1.08%	1.42%	0.61%	0.71%
***Ramachandran plot***					
Outliers (%)	0.00%	0.00%	0.00%	0.00%	0.00%
Allowed (%)	0.52%	1.34%	1.87%	1.56%	1.49%
Favored (%)	99.48%	98.66%	98.13%	98.44%	98.51%
***B factors***					
Protein	29.38	52.57	70.31	36.65	41.35
Nucleotide	30.16	52.59	52.28	29.09	42.62
Ligand	31.86	60.23	67.84	38.24	44.25

In the Mg^2+^-ATP dataset, to preserve structural features that deviate from helical symmetry, helical symmetry was applied only during the initial helical refinement to determine the filament geometry. Subsequent refinements were performed without imposing helical symmetry. In addition to the reconstruction containing density corresponding to RAD54B site 1 and site 2, this strategy enabled reconstruction of a second class containing a distinct globular density bridging two distal RAD51 protomers (*n* and *n* + 5) within the filament (Supplementary Fig. 2B, 2nd Workflow Path, Supplementary Fig. 3C). While the RAD54B N-terminal peptide binds each RAD51 protomer and therefore follows the helical symmetry of the filament (Supplementary Fig. 1B, 1st Workflow Path), the globular density within the filament groove was substantially weaker, likely reflecting conformational heterogeneity and weak interactions. To improve its reconstruction, we performed iterative rounds of focused 3D classification using a loose mask encompassing the filament groove, thereby enriching particles containing well-defined globular density (Supplementary Fig. 2B, 2nd Workflow Path). The resulting density was consistent with the AF3-predicted β-domain of RAD54B (Supplementary Fig. 2C, Supplementary Fig. 3C, D)^39^. It inserts into the groove in the filaments and induces filament compression (Supplementary Fig. 3E, F). However, due to the limited resolution in this region, detailed interaction features could not be discerned. The β-domain is located close to the ssDNA but there were no direct interactions that could be observed. We wondered whether it might contact dsDNA in the RAD51–dsDNA filaments. We obtained a 2.4 Å cryo-EM reconstruction of the RAD51–dsDNA–RAD54B-N complex in the presence of Ca²⁺-ATP (Supplementary Fig. 4, Table 1). As with the ssDNA complex, RAD54B-N binds each RAD51 protomer via the same two sites in the extreme N-terminus (Fig. 3F, Supplementary Fig. 3B). Notably, the β-domain was now clearly resolved, bridging two distal RAD51 protomers (Fig. 3F, Supplementary Fig. 3D). The AF3 model fitted into the density convincingly, with density for the loop that contacts dsDNA clearly resolved (Supplementary Fig. 3H). The β-domain contacts phosphodiester backbones of one of the two DNA strands via a positively charged loop (Fig. 3G), therefore resulting in a better resolved structure. In this structural model, there are weak electrostatic interactions between the β-domain and the two RAD51 protomers in the filament, which would stabilize and compress the filaments (Supplementary Fig. 3E–G).

Together, these cryo-EM structures reveal how RAD54B-N interacts with RAD51 filaments through three motifs and suggest that the β-domain contributes to filament stabilization and dsDNA binding, potentially providing a molecular mechanism for the enhanced recombination activity conferred by RAD54B.

### The three binding sites in RAD54B have distinct functionalities

To corroborate our structural observations and assess the functional relevance of individual RAD54B–RAD51 interfaces, we used mutagenesis and biochemical assays to dissect the contributions of the three sites. We generated several RAD54B deletion mutants: RAD54B-∆10 (removal of residues 2–10, disrupting site 1 while retaining Met1), RAD54B-∆FP (removal of residues 18-25, disrupting site 2), RAD54B-∆25 (removal of residues 2–25, disrupting sites 1 and 2), and RAD54B-∆β (removal of the β-domain, site 3) (Fig. 3A).

**Fig. 4:**
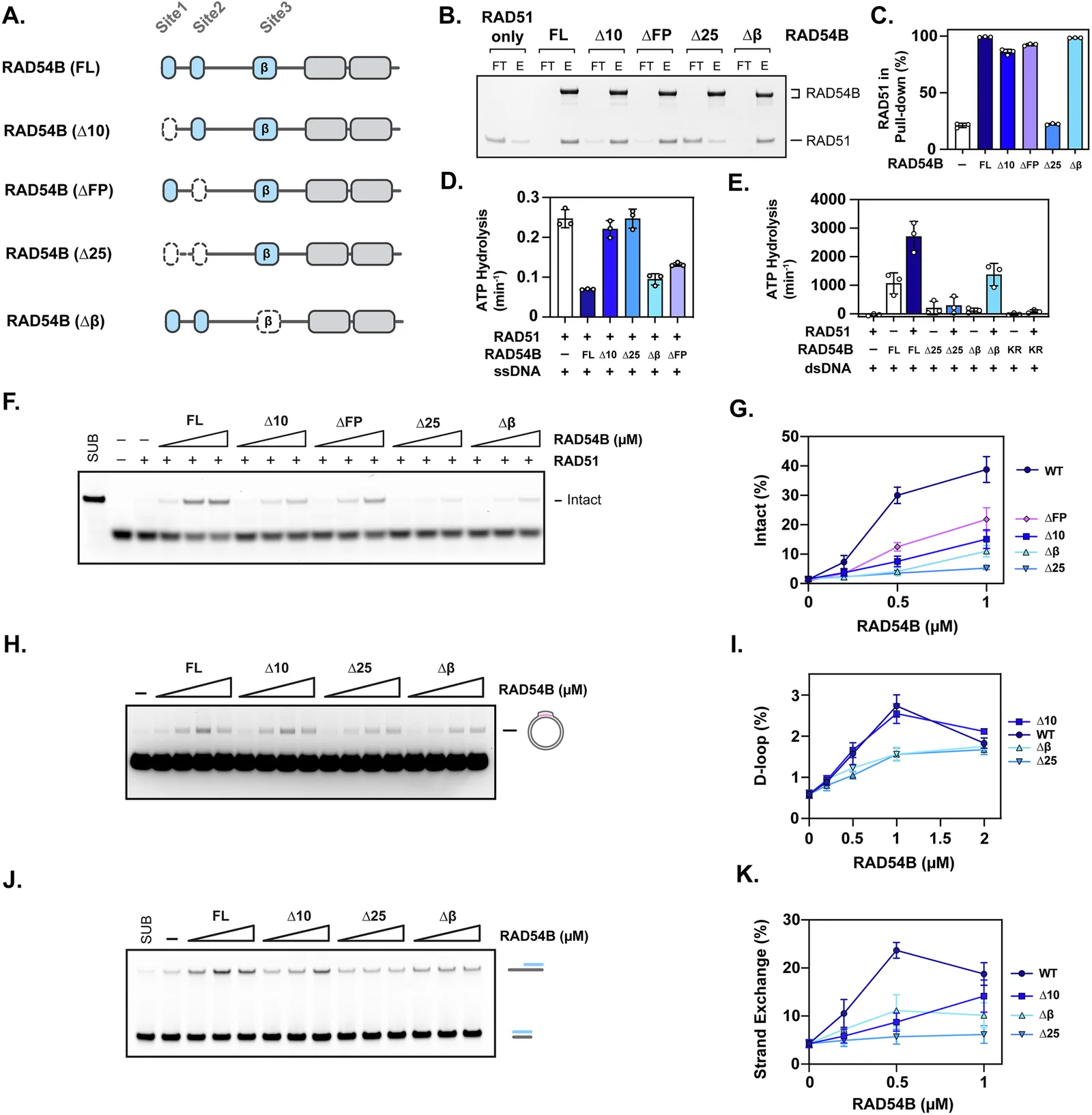
Three RAD51-binding sites in RAD54B serve distinct but complementary functions. **A** Schematics of the RAD54B mutants tested. **B** Pull-down experiment of RAD54B (full-length and deletion mutants) and RAD51. **C** Quantification of (**B**). **D** ATPase assay showing that RAD54B inhibits RAD51 ATPase activity, compared across full-length (FL) and mutants. **E** ATPase assay showing that RAD51 stimulates RAD54B-dsDNA-dependent ATPase activity, compared across full-length (FL), ∆25, and ∆β variants. RAD54B ATPase-deficient mutant (KR) as a control to support the stimulation is on RAD54. **F** ssDNA nuclease protection assay. Representative gel images showing RAD51–ssDNA filament stabilization by RAD54B (full-length and deletion mutants). **G** Quantification of (**F**). **H** Representative gel images showing the stimulation of RAD51-driven D-loop formation by RAD54B (full-length and deletion mutants) (0.2, 0.5, 1, and 2 µM). **I** Quantification of (**H**). **J** Representative gel images showing the stimulation of RAD51-driven strand exchange by RAD54B (full-length and deletion mutants) (0.2, 0.5, and 1 µM). **K** Quantification of (**J**). For (**B**–**K**), data are presented as mean ± SD from *n* independent biochemical experiments (*n* = 3).

To assess how these deletions affect RAD51 binding, we compared each mutant with full-length RAD54B. Using pull-down assays, deletion of site 1 (∆10) or site 2 (ΔFP) only mildly impaired binding, whereas removal of both sites (∆25) abolished the binding (Fig. 4A–C), suggesting that site 1 and site 2 together constitute the major binding site. In contrast, deletion of the β-domain (∆β) had no effect on RAD51 binding under these conditions, suggesting that this interaction is transient or of lower affinity, in agreement with the structural data. Together, these results indicate that RAD54B establishes its initial and dominant contact with RAD51 through sites 1 and 2, which anchors RAD54B on the filament and positions the β-domain (site 3) for subsequent engagement.

We next assessed the impact of these mutations on RAD51 ATPase activity using RAD51–ssDNA filaments. Deletion of site 1 eliminated the ability of RAD54B to inhibit RAD51 ATP hydrolysis, whereas RAD54B with site 2 deleted retained its ability to inhibit RAD51 ATPase activity. Removal of the β-domain also did not affect RAD54B-mediated inhibition of RAD51 ATPase activity significantly (Fig. 4D), indicating that site 1 is the principal determinant of RAD51 ATPase suppression by RAD54B. Conversely, adding RAD51 stimulated the ATPase activity of RAD54B when RAD54B was bound to dsDNA. The increased ATPase activity is mainly due to stimulation of RAD54B by RAD51, as the total ATPase activity remains negligible when RAD51 is added to the KR mutant of RAD54B, which is defective in ATP hydrolysis (Fig. 4E). Interestingly, both Δ25 and ∆β resulted in defective ATPase, suggesting both domains in the NTD are important for dsDNA-dependent ATPase activity of RAD54B. Adding RAD51 partially rescued ATPase activity of RAD54-∆β but not RAD54-Δ25 (Fig. 4E), suggesting that RAD51 could partially substitute for the stimulatory roles of RAD54-N on RAD54B ATPase activity, and this stimulation requires direct interactions between RAD51 and RAD54B-N, via the interaction sites identified here (Fig. 4C).

We then examined the consequences of these mutations for filament stabilization. Deletion of site 1 (∆10) or site 2 (ΔFP) markedly impaired stabilization of RAD51–ssDNA filaments (Fig. 4F, G). Site 1 is positioned adjacent to the ATP-binding pocket, offering an explanation for its inhibitory effect on ATP hydrolysis, which would also stabilize filaments. This mechanism is consistent with the increased filament stability observed when ATP hydrolysis is suppressed by substitution of Mg²⁺ with Ca²⁺ to form non-hydrolyzable Ca²⁺–ATP complexes^40^. Furthermore, site 1 inserts into the RAD51 N-terminal domain at the protomer–protomer interface, suggesting that this interaction could contribute directly to stabilization of filament architecture. In contrast, site 2 does not contribute to RAD51 ATPase inhibition, indicating that it stabilizes RAD51 filaments through an ATPase-independent mechanism. Structurally, site 2 binds across the ATPase domain along the filament (Fig. 3D), raising the possibility that it could contribute to filament stabilization by preventing dissociation. As expected, deletion of both site 1 and site 2 (∆25) eliminated filament stabilization (Fig. 4F, G). Notably, although deletion of the β-domain had minimal effects on RAD51 binding and ATPase inhibition, it severely compromised filament stabilization, consistent with a structural role in bridging adjacent RAD51 protomers and compressing RAD51 filaments (Supplementary Fig. 3E–G).

We next evaluated how these mutations affect RAD51-mediated recombination. Despite its key roles in ATPase inhibition and filament stabilization, site 1 was dispensable for stimulating stable D-loop formation under the experimental conditions: full-length RAD54B and RAD54B-∆10 supported comparable levels of D-loop formation (Fig. 4H, I). In contrast, deletion of the β-domain (∆β) reduced D-loop stimulation. We then examined strand exchange using oligonucleotide-based assays. Deletion of site 1 reduced strand exchange stimulation and shifted the dose–response to higher RAD54B concentrations (Fig. 4J, K), consistent with binding defects. Deletion of both site 1 and site 2 abolished strand exchange stimulation, while deletion of the β-domain also reduced maximal activity.

Together, these results indicate that all three sites contribute to filament stabilization and strand exchange, whereas the β-domain appears to play an important role in D-loop formation. Site 1 and site 2 promote filament stabilization via direct binding, therefore potentially strengthening protomer–protomer interactions and preventing dissociation, while site 1 also inhibits RAD51 ATPase activity, which further leads to filament stabilization. The β-domain (site 3) likely engages RAD51 filaments and promotes filament stabilization by strengthening remote RAD51 protomer–protomer contacts.

### The β-domain of RAD54B plays key roles in homologous recombination via dsDNA binding and bridging distal RAD51 protomers

**Fig. 5:**
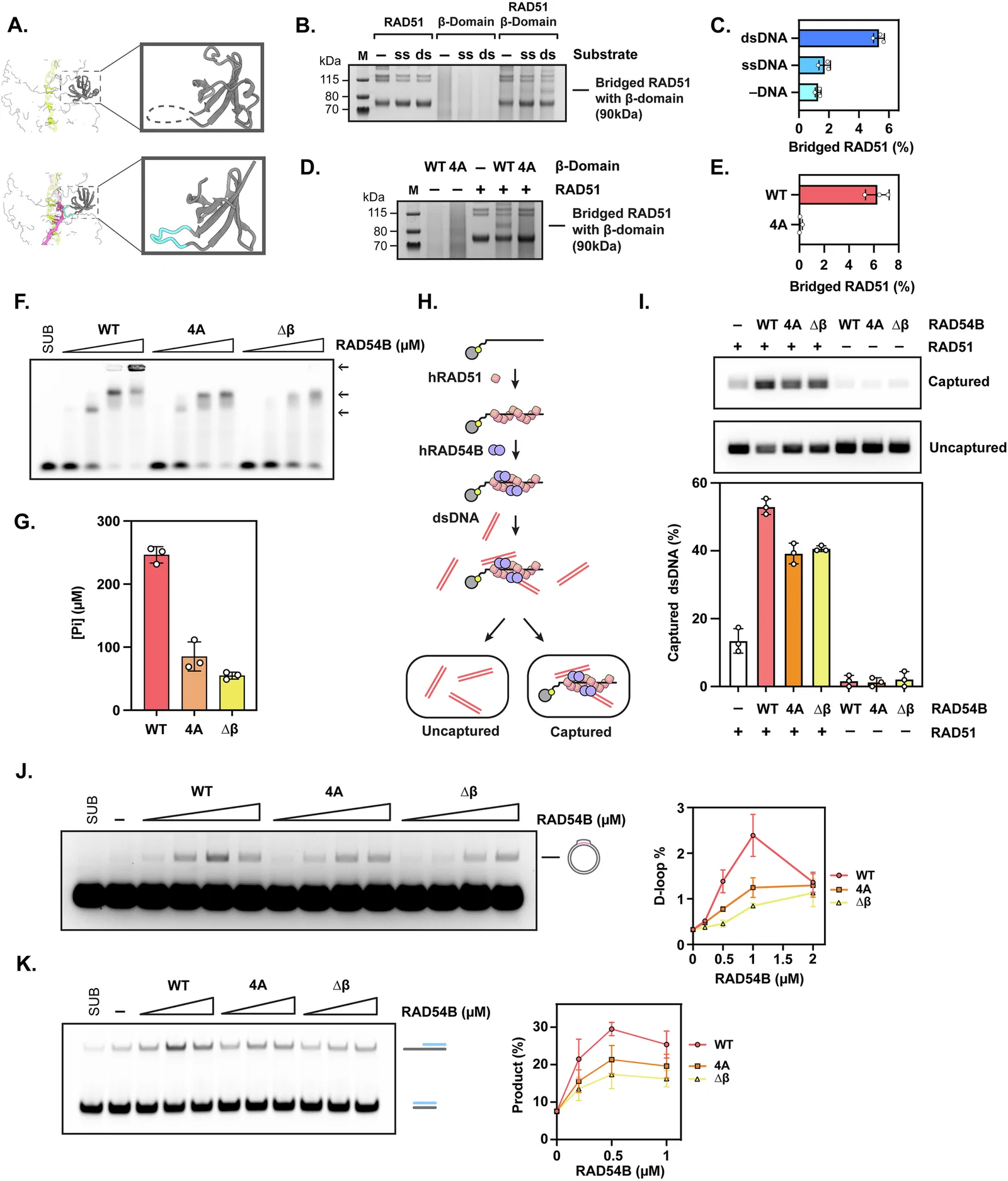
DNA-binding activity of the RAD54B β-domain is essential for maintaining intrinsic activity and bridging distal RAD51 protomers. **A** Comparison of the β-loop in the β-domain when bound to RAD51–ssDNA versus RAD51–dsDNA filaments. **B** Representing the image gel of the crosslinking experiment. RAD51, the β-domain, and mixtures of both in the absence or presence of ssDNA or dsDNA. Gel image cropped from Fig. S5C. **C** Quantification of (**B**). **D** Crosslinking experiment of wild-type and 4A mutant β-domain with RAD51 in the presence of dsDNA. Gel image cropped from Fig. S7D. **E** Quantification of (**D**). **F** Electrophoretic mobility shift assay (EMSA) showing binding of wild-type and mutant RAD54B (0.5, 1, 2, and 3 µM) to fluorophore-labeled 35-mer dsDNA. **G** ATPase assay comparing intrinsic ATPase activity of RAD54B (wild-type and mutants) in the presence of dsDNA. **H** Schematic of the dsDNA capture assay used to assess RAD51-mediated dsDNA capture. **I** Representative gel images showing stimulation of RAD51-dependent dsDNA capture by RAD54B (wild-type and mutants). **J** Representative gel images showing the stimulation of RAD51-driven D-loop formation by RAD54B (wild-type and mutants) (0.2, 0.5, 1, and 2 µM). **K** Representative gel images showing the stimulation of RAD51-driven strand exchange by RAD54B (wild-type and mutants) (0.2, 0.5, and 1 µM). For (**B**–**E**, **G**, **I**–**K**), data are presented as mean ± SD from *n* independent biochemical experiments (*n* = 3).

Structural analysis revealed that the β-domain of RAD54B bridges two distal RAD51 protomers within the filament, resulting in filament stabilization. Notably, a positively charged loop within the β-domain (β-loop) was well resolved in the RAD51–dsDNA complex but was absent in the ssDNA-bound structure (Fig. 5A, Supplementary Fig. 3D). Sequence alignment showed that this loop is highly conserved across eukaryotic species, highlighting its potential functional importance (Supplementary Fig. 5A). Together, these observations suggested that the β-domain stabilizes RAD51–DNA filaments by bridging RAD51 protomers from adjacent helical turns and, in the context of dsDNA filaments, may additionally engage DNA via the β-loop (Supplementary Fig. 5B).

To test this idea, the isolated β-domain was purified and examined in crosslinking assays with RAD51 in the presence of different DNA substrates. Upon mixing the β-domain with RAD51 or RAD51–DNA complexes, several new crosslinked species were detected (Fig. 5B and Supplementary Fig. 5C). A band of approximately 90 kDa, consistent with a complex containing two RAD51 protomers and a single β-domain, was markedly enriched in the presence of dsDNA compared with ssDNA or no DNA (Fig. 5B, C). To assess whether formation of this complex depends on β-loop–DNA interactions, four charged residues within the β-loop were mutated to alanine (4A). This mutation abolished formation of the 90 kDa crosslinked species (Fig. 5D, E and Supplementary Fig. 5D) and significantly reduced dsDNA binding by the β-domain alone (Supplementary Fig. 5E), supporting a role for the β-loop in connecting distal RAD51 protomers and interacting with dsDNA within RAD51–dsDNA filaments.

Given the strong contribution of the β-domain to D-loop formation and strand exchange (Fig. 4H–K), we next asked whether its dsDNA-binding activity plays a role in the context of full-length RAD54B. Although the isolated β-domain binds dsDNA weakly, and this interaction is disrupted by the 4A mutation (Supplementary Fig. 5E), neither the 4A mutation nor deletion of the β-domain significantly altered the overall dsDNA-binding affinity of full-length RAD54B (Supplementary Fig. 5F). This is consistent with the presence of other DNA-binding domains within RAD54B, including the C-terminal dsDNA translocase domain.

Despite these modest effects on bulk dsDNA binding, EMSA results revealed qualitative differences in complex formation. Full-length RAD54B formed three distinct dsDNA-bound species with a 35 bp substrate (Fig. 5F), suggestive of higher-order assemblies. Both the 4A mutant and the β-domain deletion reduced the highest-molecular-weight species, indicating that the β-domain contributes to higher-order RAD54B–dsDNA assemblies. Both mutations, although they do not affect dsDNA-binding affinity (Supplementary Fig. 5F), caused a pronounced reduction in dsDNA-dependent ATPase activity (Fig. 5G). These data suggest that the β-domain, including its dsDNA-binding property, might regulate higher-order assembly and ATPase activity.

Given that the β-domain can bind to dsDNA, we next tested whether the β-domain contributes to RAD54B-mediated bridging between RAD51 presynaptic filaments and dsDNA. In dsDNA capture assays, RAD51–ssDNA filaments immobilized on magnetic beads captured low levels of non-homologous dsDNA on their own, whereas addition of RAD54B substantially enhanced dsDNA capture (Fig. 5H, I). This enhancement was reduced by approximately 25% in both the 4A mutant and the β-domain deletion mutant, indicating that the β-domain, and particularly the β-loop, contributes to RAD51-mediated dsDNA engagement.

Consistent with these defects, both the 4A mutant and the β-domain deletion markedly impaired RAD54B stimulation of D-loop formation and strand exchange (Fig. 5J, K). Further structural superposition of the RAD54B–RAD51–dsDNA complex with recently reported RAD51–D-loop structures^34,41,42^ places the β-domain adjacent to the heteroduplex DNA within the D-loop (Supplementary Fig. 5G, H). Together with the biochemical data, these observations support a role for the β-domain in promoting RAD51 filament conformational transitions from presynaptic filaments (ssDNA-bound) to homologous pairing and to synaptic filaments (D-loop intermediates).

### The RAD51 interaction sites and the β-domain of RAD54B are important for DSB repair by HR in U2OS cells

**Fig. 6:**
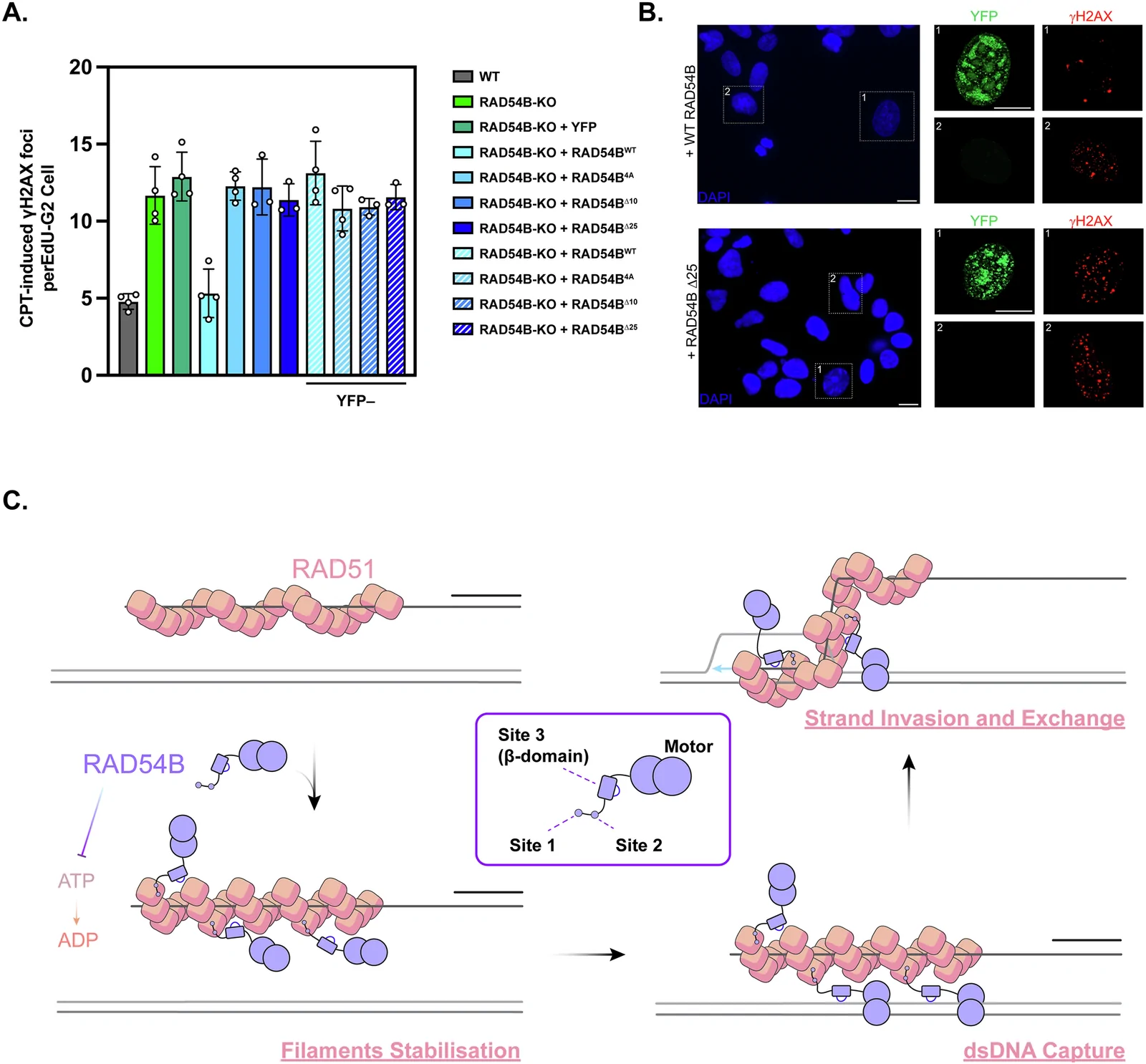
The RAD51 interaction sites and the β-domain of RAD54B are important for DSB repair by HR in U2OS cells. **A** γH2AX foci levels in U2OS WT and RAD54B-KO cells treated with 5 nM Camptothecin (CPT) for 24 h. RAD54B-KO cells were left untransfected or were transiently transfected with constructs for YFP-only, YFP-tagged RAD54B WT, RAD54B4A, RAD54BΔ10, or RAD54BΔ25. 1 h before fixation, 10 µM EdU was added to monitor the cell cycle. γH2AX foci were quantified in EdU-negative G2-phase cells. In transfected cells, γH2AX foci were enumerated in YFP-positive (solid bars) and YFP-negative cells (striped bars). Spontaneous foci were subtracted (see Supplementary Fig. 7). Bars represent the mean ± SEM from n independent experiments (*n* = 3 for RAD54BΔ10 and RAD54BΔ25; *n* = 4 for all other conditions). Dots represent individual experiments. **B** Representative IF images of γH2AX foci in cells treated with CPT. Images are representative of the independent experiments quantified in (**A**). Overview images show the DAPI signal obtained from the scanning microscope. Cell nuclei with a G2 DNA content negative for EdU (not shown) and either positive (1) or negative (2) for YFP were selected. Scalebars represent 10 µm. **C** Schematic model for RAD54B’s functionalities in HR. Upon initial engagement with RAD51–ssDNA filaments, RAD54B inhibits RAD51 ATPase, stabilizes RAD51–ssDNA filaments, priming the filament for subsequent dsDNA capture. During homology pairing, RAD54B β-domain is positioned to engage the incoming dsDNA strand. Following homology recognition, RAD54B β-domain can contribute to stabilizing the D-loop structure.

To investigate the function of RAD54B in cells and the importance of the sites we identified, we generated U2OS RAD54B-KO cells using CRISPR/Cas9 and validated them by sequencing. We treated exponentially growing cell cultures for 24 h with the topoisomerase I inhibitor camptothecin (CPT). CPT induces single-stranded lesions, which are converted during replication into DSBs. Following the progression of cells into G2 phase, such DSBs are repaired by HR^43^. Hence, we scored the level of γH2AX foci as a marker for DSBs in G2-phase cells as previously described^44^. RAD54B-KO cells showed markedly elevated foci numbers compared with WT cells, indicating the importance of RAD54B for repairing CPT-induced DSBs (Fig. 6A and Supplementary Fig. 6A). This finding is consistent with elevated γH2AX foci levels in RAD54B-deficient ovarian cancer cells treated with a PARP inhibitor^45^, which, similar to CPT, induces DSBs during S phase^46^. We then transiently transfected U2OS RAD54B-KO cells with YFP-tagged WT or mutated RAD54B constructs and assessed foci levels after CPT treatment in YFP-positive G2-phase cells (Fig. 6A). The expression of WT RAD54B reduced the foci levels of RAD54B-KO cells to those of WT cells. As a control, we analyzed YFP-negative G2-phase cells in the same samples, which did not exhibit reduced foci numbers (Fig. 6A, B and Supplementary Fig. 6A). In strong contrast to the expression of WT RAD54B constructs, the expression of RAD54B constructs carrying a RAD54B4A, a RAD54B Δ10 or a RAD54B Δ25 mutation (Table 2) failed to reduce foci numbers in RAD54B-KO cells (Fig. 6A, B and Supplementary Fig. 6A, B). Notably, the mutant proteins have similar expression levels and distributions as judged by YFP signals and images (Supplementary Fig. 6B). These results show that the two RAD51 interaction sites, as well as the β-domain, are important for RAD54B function in human cells.

**Table 2:** primer sequences to make mutants used in cell biology experiments

Primer	Forward (fwd) sequence (5′−3′)	Reverse (rev) sequence (5′−3′)
RAD54B4A	GCCTAGTTAAATATTTCAGTGTT-GTTTGGTGTAAGCCTTCAGCCG-CTGCTGCGAAAAAGTGGGAAG-GTGATGCTGTTCTTATTGTAAAAGGAA	TTCCTTTTACAATAAGAACAGC-ATCACCTTCCCACTTTTTCGCA-GCAGCGGCTGAAGGCTTACAC-CAAACAACACTGAAATATTTAAC-TAGGC
RAD54B Δ10	CAGGCTGCCACCATGCAGGGGAAT-TCCTTC	GAAGGAATTCCCCTGCATGGTGGCA-GCCTG
RAD54B Δ25	CAGGCTGCCACCATGAGTAATCC-AGGTCTGAATG	CATTCAGACCTGGATTACTCATGGT-GGCAGCCTG

## Discussion

### Unique contributions of the three sites and mechanisms of HR modulation

Our data presented here reveal that RAD54B acts as a multifunctional modulator that bridges RAD51 and dsDNA, stabilizing recombination intermediates and coordinating the transition from filament formation to strand invasion. The NTD mediates critical interactions with RAD51 and dsDNA, while the ATPase domain drives the remodeling necessary for D-loop formation (Fig. 6C). The three sites identified in our structures have distinct contributions during HR. Binding experiments identify site 1 and site 2 as being responsible for recruiting RAD54B to RAD51–ssDNA filaments. Site 1 also inhibits RAD51 ATPase activity, while all three sites contribute to filament stabilization. ATPase inhibition stabilizes RAD51–DNA filaments. However, filament stabilization does not necessarily result from ATPase inhibition as demonstrated by site 3, which stabilizes the filaments by bridging two distal RAD51. Indeed, filaments can be stabilized by enhancing DNA binding and RAD51 protomer–protomer interactions. Inhibiting ATP hydrolysis stabilizes DNA binding, as the ADP-bound form does not stably engage with DNA^33^. Site 1 interacts with RAD51 N-domain, which is located at the protomer–protomer interface, therefore likely contributes to protomer–protomer interactions in addition to inhibiting ATP hydrolysis. Site 2 likely contributes to filament stabilization via preventing filament dissociation, while site 3 contributes to filament stabilization through bridging distal protomers and filament compression, which would also increase the protomer–protomer interface. In the dsDNA filament, site 3 also stabilizes filaments via contacting dsDNA.

Since all sites contribute to strand exchange, mirroring the filament stability assays but site 3 also plays important roles in D-loop formation, highlighting the differences in these two assays. Indeed, in oligonucleotide assays, strand invasion will promptly proceed to strand exchange, while stable D-loop formation requires strand invasion, D-loop propagation, and D-loop stabilization due to the torsional constraint imposed by plasmid DNA. In strand exchange assays, stable filaments are key to allow homologous DNA pairing, and indeed, strand exchange activities largely mirror that of filament stabilization (Fig. 4G, K). In D-loop assays, deleting site 1 has no effect, whereas deleting the β-domain, although it has no effect on RAD51 binding or RAD51 ATPase activity, is defective (Fig. 4I). Given that the β-domain interacts with dsDNA and stimulates RAD51–ssDNA dsDNA capture, it raises the possibility that this region can contribute to donor dsDNA engagement during the homology search. Moreover, overlaying the structure of RAD54B in complex with RAD51–dsDNA presented here with recently reported RAD51-D-loop^34,41,42^ structures places the RAD54B β-domain adjacent to the heteroduplex DNA within the D-loop (Supplementary Fig. 5G, H). Together, these properties could contribute to its roles in D-loop stabilization and strand exchange.

Intriguingly, both the first 25 amino acids and the β-domain are essential for the dsDNA-dependent ATPase activity of RAD54B (Fig. 4E). The ability of β-domain binding to dsDNA may help regulate the productive association of RAD54B with dsDNA, stimulating ATPase activity, and this function can be partially replaced via RAD51 binding as RAD51 binding can restore RAD54B-∆β ATPase activity to some degree (Fig. 4E). The first 25 amino acids are the main RAD51-binding site and how it regulates RAD54B ATPase activity is currently unclear (Fig. 4E).

Together our data show that RAD54B promotes RAD51 recombinase activities during several key stages via distinct structural domains (Fig. 6C): binds to RAD51–ssDNA via the first two sites, inhibiting RAD51 ATPase activity (site 1), and stabilises RAD51–ssDNA filaments (all three sites), promotes dsDNA capture (site 3 is positioned to bind to donor dsDNA), stimulates RAD54B-dsDNA-dependent ATPase activity (all three sites) and stabilize D-loop structure (site 3 could contribute to this).

### Binding site differences and functional distinctions between RAD54 and RAD54B

Human RAD54 and RAD54B, together with their yeast orthologues Rad54 and Rdh54, have been proposed to perform overlapping functions during homologous recombination. Although genetic and biochemical studies suggest that these proteins possess both shared and distinct activities, the molecular basis underlying their functional similarities and differences remains unclear. Our study demonstrates that RAD54B employs three structurally distinct RAD51-interacting elements to regulate recombination and, unlike RAD54, does not promote dissociation of RAD51 from dsDNA.

The N-terminal RAD51-binding peptide (site 1) is highly conserved between human RAD54 and RAD54B (Supplementary Fig. 7A). Consistent with this conservation, the cryo-EM structure of the RAD54 N-terminal peptide bound to RAD51 is nearly identical to that of RAD54B, indicating that both proteins engage the same binding pocket through a conserved mechanism (Supplementary Fig. 7).

Sequence alignment of the three sites showed that only site 2 is conserved among all four proteins, while site 1 is only conserved in human proteins (Supplementary Fig. 8A–C). The recently reported structure of yeast Rad54 bound to Rad51–ssDNA filaments shows interactions that overlap with the binding sites of both site 1 and site 2 identified here (Supplementary Fig. 8D, E)^12^. Although the site 2 interaction is highly conserved, the site 1 interface differs between human and yeast proteins, consistent with sequence divergence in this region. Given the strong conservation of the corresponding sequence between yeast Rad54 and Rdh54, it is likely that Rdh54 engages Rad51 through a similar mechanism.

In contrast, the β-domain (site 3) is conserved only in RAD54B and Rdh54 (Supplementary Fig. 8C). Our biochemical and structural analyses demonstrate that this domain contributes to dsDNA interaction, RAD54B ATPase activity, RAD54B–dsDNA complex formation, and stimulation of strand invasion and D-loop formation. We therefore propose that acquisition of the β-domain represents a key evolutionary divergence that underlies the distinct biochemical and functional properties of RAD54B (and Rdh54) relative to RAD54 (and Rad54).

Intriguingly, unlike RAD54, yeast Rad54 and Rdh54, RAD54B is unable to dissociate RAD51 from dsDNA (Fig. 1I)^25,35,36^. It will be interesting to see if this is related to the unique sites we identified here, particularly site 3, which is important for dsDNA-binding and ATPase activity. Both human RAD54 and yeast Rad54 lack the β-domain, while yeast Rad54 and Rdh54 lack site 1, which inhibits RAD51 ATPase activity. However, the exact molecular basis of these differences requires further comparative biochemical and structural studies of these proteins. Recently, RAD54 and RAD54B have been shown to play important roles in distinct HR sub-pathways, with RAD54 primarily involved in the double-Holliday junction resolution pathway while RAD54B is involved in synthesis-dependent strand annealing^24^. Further, while both RAD54 and RAD54B deletions result in RAD51 foci accumulation, only RAD54 deletions result in elongated RAD51 foci (Supplementary Fig. 6C, D), suggesting that they might play key roles at different stages of HR. Which aspect of the two proteins, whether the differences in their ability to remove RAD51 from dsDNA and whether the β-domain contributes to their distinct functionalities, remains to be investigated.

### RAD51 modulators use shared interaction sites on RAD51

The RAD51 surface where site 1 and site 2 bind is a highly conserved interaction site that is also shared by several other RAD51 regulators, suggesting that these regions constitute a major regulatory interface on RAD51 (Supplementary Fig. 8D)^12,33^. Consistent with this idea, our recent work on RAD51AP1 demonstrated that RAD51AP1 also binds to where site 1 binds and extends further into a deep cleft on the RAD51 surface^33^. Similar to RAD54B site 1, this site on RAD51AP1 is also important in inhibiting RAD51 ATPase activity and stabilizing RAD51 filaments (Supplementary Fig. 8D). Yeast Hed1 and yeast Rad54 engage at the same RAD51 surface as RAD51AP1 including the extended interactions in the deep cleft (Supplementary Fig. 8D). Site 2 constitutes FxxP motif, which is present in multiple RAD51 modulators, including the C-terminal TR2 motif of BRCA2, RAD51AP1, yeast Rad54, Rdh54 and FIGNL1 (Supplementary Fig. 8E)^12,33,46–49^. In the context of BRCA2 TR2, the FxPP motif has been shown to alter RAD51 dimer conformation and promote preferential binding to dsDNA^48^. In contrast, our structures do not reveal substantial changes in RAD51 protomer interfaces upon RAD54B binding, suggesting that this motif can engage RAD51 in filamentous state without inducing major conformational rearrangements.

The shared sites among RAD51 modulators on RAD51 suggest a model for how antagonists such as yeast Rad54 and Hed1 regulate each other^12^. However, it also raises the question of how modulators that have overlapping activities might compete with each other for RAD51 binding. Yeast Rad54 and Rdh54 are proposed to act synergistically on RAD51. Given that they share sites 1 and 2, which are the primary RAD51-binding sites, they will either compete with each other or bind to different RAD51 molecules within the filaments. It will be interesting to see how RAD54 and RAD54B, and indeed with other modulators, together interact and act on RAD51.

In addition to the shared sites, the regulators also use unique sites. For example, RAD54B (and yeast Rdh54) contains a unique β-domain that plays multiple roles in regulating RAD51 activities and the HR process. FIGNL1, on the other hand, is the only regulator shown so far to interact with RAD51 N-terminus and dissociate RAD51 by pulling on the N-terminal peptide. These versatile binding sites and modes of action likely give rise to their unique properties^49^, allowing these RAD51 modulators to perform distinct functions during HR.

## Methods

### Sequence alignment

A multiple sequence alignment of RAD54B sequences of representative species was generated using Clustal Omega^50^.

### Cloning

The Human RAD54B with a C-terminal Twin-Strep (TS) or 10xHis-Twin-Strep tag sequence was synthesized from IDT, amplified by PCR, and inserted into a pFBDM vector. Substitution and truncations were generated by PCR, and the fragments were combined using the NEBuilder HiFi assembly kit (NEB).

### Protein purification

Human RAD51 was cultured and purified as previously described^33^. RAD51^C319S^ was cultured and purified in the same way as the wild-type protein. To label the RAD51^C319S^, 15 µL of AuroraFluor 488 (Biosynth Laboratories Ltd, 25 mM in DMSO) was incubated with 1.3 mL RAD51^C319S^ at 1.12 mg/mL at 4 °C overnight. Then, further purification was carried out on a HiTrap Heparin HP column in 20 mM Tris [pH 7.5], 150–2000 mM NaCl, 0.5 mM TCEP.

The full-length RAD54B mutants and RAD54-N/C proteins used in most of the studies (see below) were expressed in baculovirus expression systems either with a Twin-Strep tag or with a GST tag. Twin-Strep-tagged proteins were infected Hi5 cells. Hi5 cells (density 1 × 10^6^) were infected with the recombinant baculovirus at a multiplicity of infection of 2, and after 48 h of incubation, were collected by centrifugation (1000 × *g*). Cell pellets were resuspended in lysis buffer (20 mM HEPES [7.5], 300 mM KCl, 0.5 mM TCEP, 10% glycerol) supplemented with cOmplete protease inhibitors (Roche^TM^). Cells were lysed by sonication and clarified by centrifugation before applying to a 5 mL StrepTrap HP column (Cytiva^TM^). The column was washed with 50 mL lysis buffer and eluted in 50 mL elution buffer (20 mM HEPES [7.5], 200 mM KCl, 0.5 mM TCEP, 10% glycerol, 10 mM desthiobiotin). The protein was further purified using a 5 mL Heparin HP column (Cytiva^TM^) run in RA buffer (20 mM Tris [pH 7.5], 0–1 M KOAC, 0.5 mM TCEP, 10% glycerol). The peak fractions were pooled and concentrated to 0.5 mL before loading to a Superdex 200 increase 10/300 column (Cytiva^TM^) equilibrated with RA150 buffer (20 mM Tris [7.5], 150 mM KOAC, 0.5 mM TCEP, 10% glycerol). Finally, the proteins were concentrated and frozen in aliquots in liquid nitrogen.

For RAD54, RAD54K189R, RAD54B, and RAD54BK332R proteins used in RAD51 displacement assays and ATPase assays (Fig. 4E), the proteins were produced with a GST tag and a TEV protease site added between the GST tag and the start codon of each protein. The recombinant proteins were expressed using the Baculovirus Expression System (Thermo Fischer Scientific) by infecting SF9 insect cells with the P3 generation of respective viruses for 48 h. Post infection, the cells were centrifuged and the pellets were washed twice with ice-cold PBS before freezing and storing them in −80 °C. The purifications were done at 4 °C by lysing the cell pellets in lysis buffer [50 mM Tris-HCl, pH 7.5, 1 M KCl, 2 mM EDTA, 0.5% IGEPAL CA-630, 0.5 mM TCEP, 10% glycerol supplemented with Roche cOmplete protease inhibitor cocktail (Millipore Sigma)] using a Dounce homogenizer for mechanical cell lysis. This was followed by 30 min of stirring of homogenized cells. The crude cell lysates were ultracentrifuged (35,000 RPM for 1 h) to remove cell debris. The clarified lysate was loaded on lysis buffer-equilibrated glutathione agarose beads (Thermo Fischer Scientific) and incubated for 4 h with gentle agitation to allow protein immobilization on beads. Post incubation, the mix was transferred to gravity flow chromatography columns and beads were washed with 10 column volumes (CV) of High salt buffer (20 mM Tris-HCl, pH 7.5, 1 M KCl, 1 mM EDTA, 0.5% IGEPAL CA-630, 0.5 mM TCEP, 10% glycerol supplemented with protease inhibitor cocktail) followed by wash with 10 CV of Low Salt buffer (20 mM Tris-HCl, pH 7.5, 400 mM KCl, 1 mM EDTA, 0.5% IGEPAL CA-630, 0.5 mM TCEP, 10% glycerol supplemented with protease inhibitor cocktail). In-column digestion by GST-TEV protease (600 µg) was performed overnight to cleave the GST tags from the recombinant proteins. The proteins were eluted, analyzed, and concentrated to a 2 ml volume. The concentrated proteins were then loaded onto low salt buffer-equilibrated Superdex 10/300 GL size exclusion columns (Cytiva). The eluted fractions were analyzed on SDS-PAGE gels to test purity; following this, the fractions were concentrated, flash frozen in liquid nitrogen, and stored in −80 °C in small aliquots. Quality control tests were performed on purified proteins to test purity, homogeneity, and contamination with DNA or nucleases^51^.

RAD54B beta-domain (wild-type and the mutant) was expressed from Hi5 cells with an N-terminal 6xHis tag and a C-terminal Twin-Strep tag. RAD54B beta-domain (WT) purified in a similar scheme as RAD54B Full-length purification, except the gel filtration column is Superdex 75 increase 10/300 (Cytiva^TM^). RAD54B beta-domain (4A mutant) is initially bound and eluted from a 5 mL StrepTrap HP column. Then, loaded on a 5 mL HisTrap Q column (Cytiva^TM^). The flow-through from Q column is collected before applying to a 5 mL HisTrap HP column (Cytiva^TM^) equilibrated with H150 buffer (25 mM HEPES [7.5], 150 mM NaCl, 5% glycerol, 0.5 mM TCEP), then eluted in 40 mL H150 supplemented with 300 mM imidazole. The proteins were concentrated and gel-filtrated with a Superdex 75 increase 10/300 column.

### Pull-down assays

Twin-Strep- or 10×His-Twin-Strep-tagged RAD54B (1 µM) in 30 µL of pull-down buffer (30 mM Tris-OAc [pH 7.5], 100 mM KCl, 2 mM ATP, 0.1% Tween-20, 1 mM TCEP, and 5% glycerol) was immobilized on Strep-Tactin Sepharose High Performance resin (Cytiva™) by incubation at 25 °C for 30 min with mixing. RAD51 or RAD51 (C319S)-AF488 (1 µM) was then added and incubated for an additional 30 min with mixing. The supernatant containing unbound RAD51 was collected, and bound proteins were eluted by incubation with SDS-PAGE loading buffer (65 °C, 1300 rpm, 10 min). Protein content in both fractions was analyzed by SDS-PAGE, and gels were imaged using a Bio-Rad ChemiDoc MP Imaging System™. Band intensities were quantified using FIJI. Images were background-subtracted using the rolling-ball method (50 pixels), and the intensities of RAD51 and RAD54B bands in the supernatant and eluate were measured. The percentage of protein recovered in the eluate was then calculated.

### ssDNA nuclease protection assay

A Cy5-labeled oligonucleotide (60-mer, BA586; 10 µM nt) in ssDNA nuclease protection buffer (25 mM Tris-OAc [pH 7.5], 100 mM KCl, 2 mM ATP, 5 mM MgCl₂, 0.5 mM TCEP, 2.5% glycerol) was incubated with RAD51 (1 µM) at 37 °C for 5 min prior to addition of the indicated RAD54B. After a further 5 min incubation at 37 °C, DNase I (4 units; NEB™ M0303) was added, and reactions were incubated for 10 min. Reactions were stopped by the addition of deproteinization buffer (final concentrations: 12 mM Tris-Cl [pH 7.5], 7.5 mM EDTA, 0.45% SDS, 0.75 mg/mL proteinase K). DNA products were resolved on 10% PAGE gels and imaged using a Bio-Rad ChemiDoc MP Imaging System™. Quantification was performed in FIJI (Schindelin et al., 2012). Images were background-subtracted using the rolling-ball method (50 pixels), and the total lane signal was measured. The fraction of signal corresponding to intact DNA was expressed as a percentage of the total.

### dsDNA nuclease protection assay

A Cy5-labeled oligonucleotide (213-mer, BA713; 5 µMbp) in dsDNA nuclease protection buffer (25 mM Tris-OAc [pH 7.5], 50 mM KCl, 2 mM ATP, 5 mM MgCl₂, 0.5 mM TCEP, 2.5% glycerol) was incubated with RAD51 (0.5 µM) at 37 °C for 5 min prior to addition of the indicated RAD54B. After a further 5 min incubation at 37 °C, DNase I (2 units; NEB™ M0303) was added, and reactions were incubated for 10 min. Reactions were stopped by the addition of deproteinization buffer (final concentrations: 12 mM Tris-Cl [pH 7.5], 7.5 mM EDTA, 0.45% SDS, 0.75 mg/mL proteinase K). DNA products were resolved on 2% agarose gels and imaged using a Bio-Rad ChemiDoc MP Imaging System™. Quantification was performed in FIJI. Images were background-subtracted using the rolling-ball method (50 pixels), and the total lane signal was measured. The fraction of signal corresponding to intact DNA was expressed as a percentage of the total.

### D-loop assay

RAD51 (1 µM) was incubated with a 90-mer ssDNA oligonucleotide (BA717; 6 µMnt) in D-loop buffer (25 mM Tris-OAc [pH 7.5], 50 mM KCl, 2 mM ATP, 2 mM MgCl₂, 0.5 mM CaCl₂, 0.5 mM TCEP, 0.1 mg/mL BSA, 3 mM phosphocreatine, 20 u/mL CPK) at 37 °C for 5 min. The reaction was initiated by adding the indicated RAD54B and pBlueScript (pBS-SK-II; 120 µM nt). Reactions were incubated at 37 °C for 10 min, deproteinized, resolved by PAGE, and imaged as described above. Quantification was performed using FIJI^52^. Images were background-subtracted using the rolling-ball method (50 pixels), and the total lane signal was measured. The fraction of the signal corresponding to the product was expressed as a percentage of the total. For each experiment, the value for the substrate alone was subtracted from all samples.

### Strand exchange assay

RAD51 (1 µM) was incubated with a 116-mer ssDNA oligonucleotide (BA494; 3 µM nt) in strand exchange buffer (25 mM Tris-OAc [pH 7.5], 50 mM KCl, 2 mM ATP, 1 mM MgCl₂, 2 mM CaCl₂, 0.5 mM TCEP, 0.1 mg/mL BSA) at 37 °C for 7 min. RPA (0.2 µM) was then added, and after an additional 7 min incubation at 37 °C, the reaction was initiated by adding the indicated RAD54B and 60-mer dsDNA (BA586-BA504; 1 µM bp). Reactions were incubated at 37 °C for 10 min, deproteinized, resolved by PAGE, and imaged as described above. Quantification was performed using FIJI^52^. Images were background-subtracted using the rolling-ball method (50 pixels), and the total lane signal was measured. The fraction of the signal corresponding to the product was expressed as a percentage of the total. For each experiment, the value for the substrate alone was subtracted from all samples.

### ATPase assay

All the ATPase assays were conducted using a malachite green reagent-based spectrophotometric method^53^. This is a sensitive method for the detection of released phosphate, in which a green complex formed between Malachite Green, molybdate, and free orthophosphate is measured on a spectrophotometer at 620 nm.

RAD51 (3 µM) and poly-dT ssDNA (BA622, 72-mer; 9 µM nt) were incubated in ATPase buffer (35 mM Tris-OAc [pH 7.5], 50 mM KCl, 0.5 mM ATP, 2 mM MgCl_2_, 1 mM TCEP, 5% glycerol) supplemented with RAD54B (3 µM) at 37 °C for 60 min. Reactions were stopped by the addition of 50 mM EDTA and diluted two-fold to reduce phosphate concentration. Absorbance was measured at 620 nm using a CLARIOstar plate reader, and phosphate concentration was determined with a commercial colorimetric kit (MAK307, Merck) according to the manufacturer’s instructions.

RAD54B intrinsic ATPase activity was measured by incubating 150 nM RAD54B and 30 µM nt pBS-SK-II plasmids in ATPase buffer but with 0.25 mM ATP (37 °C, 10 min). Reactions were stopped by the addition of 50 mM EDTA and diluted 20-fold to reduce phosphate concentration. Absorbance was measured at 620 nm using a CLARIOstar plate reader, and phosphate concentration was determined with a commercial colorimetric kit (MAK307, Merck) according to the manufacturer’s instructions.

To measure the effects of RAD51^54^ on RAD54B ATPase activity, ATP hydrolysis assays were performed using a two-solution reaction setup. Solution I contained 25 mM HEPES (pH 7.5), 7 mM MgOAc₂, 1 mM DTT, 30 µg/mL BSA, 0.1% CHAPS, 2 mM ATP, with or without ΦX174 RFI dsDNA (12 µM bp; New England Biolabs) and with or without RAD51 (300 nM). Solution II contained the same buffer components and ATP concentration, with or without RAD54B (20 nM; full-length or mutant variants). Ten microliters of Solution I containing dsDNA with or without RAD51 (1 RAD51 per 40 bp) were incubated at 37 °C for 10 min, after which 10 µL of Solution II containing RAD54B full-length or mutant proteins (Δ25 or Δβ-domain; 1 RAD54B per 600 bp or 1 RAD54B per 15 RAD51 molecules) was added and incubated for an additional 10 min at 37 °C. Reactions were developed by adding 6 µL of malachite green reagent (1.6% ammonium molybdate, 0.16% malachite green, 4.26 M HCl, 1.07% polyvinyl alcohol), followed immediately by quenching with 6 µL of 6% sodium citrate, bringing the final reaction volume to 32 µL. Absorbance was measured at 620 nm using a SpectraMax iD5 plate reader (Molecular Devices). Reactions lacking protein were used as blank and negative controls. ATP hydrolysis rates were calculated using the formula “Rate of ATP hydrolysis = [Fraction of normalized values (NV) × [ATP] (nM)/ time of reaction (min − 1)/ concentration of protein(nM)]”.

### RAD51 displacement assays from dsDNA

A magnetic-bead-based assay was designed to test RAD51 displacement by RAD54B^55^. 5′-Biotinylated 700 base pairs dsDNA substrate was generated by PCR amplification of PhiX174 RFI (NEB) using a non-biotinylated forward primer with a 5′Biotinylated reverse primer. The biotinylated dsDNA was immobilized on streptavidin magnetic beads (Thermo Fischer Scientific), quality control assessment was performed, and the concentration was determined to be 51.2 nM molecules.

The assay was performed by incubating sub-saturating amounts of HsRAD51 (2.5 µM; 1 RAD51 per 5 base pairs) with magnetic bead-immobilized Biotin-700 bps dsDNA (12.6 µM base pairs) in 18 µl with buffer containing 35 mM Tris-Acetate (pH 7.4), 50 mM KCl, 1 mM DTT, 250 µg/ml BSA, 5 mM MgCl_2_, 2 mM ATP, 8 mM Phosphocreatine, 28 µg/ml Creatine Kinase. The RAD51–dsDNA complex was allowed to form for 5 min at 37 °C, followed by the addition of 200 nM of HsRAD54, HsRAD54K189R, HsRAD54B, or HsRAD54BK332R proteins in 1 µl volume. The samples were incubated at 23 °C for 3 min to allow protein loading to dsDNA but not fully activate their motor activity. Finally, a non-homologous 100-mer-ssDNA trap (150 µM nucleotides) was added to the mix in 1 µl volume to make a total reaction volume of 20 µl. The reaction was set at 30 °C for 15 min before separating the supernatant and bead fractions. The bead-immobilized proteins were eluted with 20 µl of 1% SDS, and 10 µl of each of the fractions were analyzed by SDS-PAGE and SYPRO-Orange (Thermo Fischer Scientific) staining (Fig. 1I). Stained SDS-PAGE gels were quantified using ImageLab software (Bio-Rad), and the results were plotted as percent RAD51 displaced using Graphpad Prism software (Dotmatics).

### Electrophoretic mobility shift assay

RAD51 (5 µM) and ssDNA (BA714, 65-mer; 15 µMnt) or dsDNA (BA586-504, 60-mer; 12 µMbp) were incubated in filaments EMSA buffer (25 mM HEPES-KOH [pH 7.5], 1 mM TCEP, 100 mM KCl, 5 mM MgCl₂, 2 mM ATP) at 25 °C for 10 min, followed by addition of the indicated RAD54B and incubation for 5 min. Reactions were crosslinked with 0.1% glutaraldehyde (0.1% glutaraldehyde was empirically optimized to preserve the assembled complexes while minimizing excessive crosslinking and aggregation) for 5 min and quenched with 1 M Tris-Cl [pH 7.5]. Products were resolved on 0.8% agarose gels.

The indicated RAD54B and dsDNA substrates (BA715, 35-mer; 17.5 µMbp) were incubated in binding buffer (25 mM Tris-OAc [pH 7.5], 1 mM TCEP, 100 mM KOAc, 5 mM MgOAc₂, 0.1 µg/µL BSA) at 25 °C for 10 min. Products were resolved on 0.8% agarose gels.

### RAD51-bridge crosslink assay

RAD51 (6 µM) was incubated with or without DNA substrates (BA716, 60-mer, 18 µMnt; BA503-504, 60-mer, 18 µMbp) in crosslinking buffer (25 mM HEPES-KOH [pH 7.5], 1 mM TCEP, 2 mM ATP, 5 mM CaCl₂) at 37 °C for 5 min. RAD54B β-domain (15 µM) was then added, and reactions were incubated for an additional 15 min. Crosslinking was initiated by adding 5 mM bis(sulfosuccinimidyl) suberate, a water-soluble homobifunctional NHS-ester crosslinker that reacts with primary amines (predominantly lysine side chains and protein N-termini), and incubating for 5 min at room temperature. With an 11.4 Å spacer arm, BS3 captures proteins in close spatial proximity. Products were resolved by SDS-PAGE.

### dsDNA capture assay

Biotin-labeled ssDNA (BA655, 100-mer; 5 µMnt) in capture buffer (25 mM Tris-OAc [pH 7.5], 1 mM DTT, 150 mM KOAc, 0.05% Tween-20, 2 mM ATP, 5 mM MgOAc₂, 5% glycerol) was immobilized on magnetic beads (Dynabeads™ M-280 Streptavidin) prior to the addition of RAD51 (1.67 µM) and incubation at 25 °C for 10 min with shaking. RAD54B (1 µM) was then added and incubated for an additional 5 min. The supernatant was removed using a magnetic stand, and the bead-bound complexes were resuspended in 10 µL capture buffer containing fluorophore-labeled dsDNA (BA586-504, 60-mer; 5 µMbp) and incubated with mixing at 25 °C for 30 min. The supernatant containing uncaptured dsDNA was collected, and bead-bound dsDNA was eluted by deproteinization (37 °C, 15 min, 1200 rpm). DNA from both fractions was analyzed by agarose gel electrophoresis.

### MST

RAD54B-dsDNA interaction studies (full-length or β-domain) were performed using 5′ Cy5-labeled dsDNA substrates (35-mer, BA715). Fluorescently labeled DNA in binding buffer (25 mM Tris-OAc [pH 7.5], 1 mM TCEP, 50 mM KOAc, 0.05% Tween-20) was diluted to 10 nM and mixed with an equal volume of a serial dilution of RAD54B. Samples were incubated at room temperature for 15 min before loading into MST capillaries. MST experiments were carried out on a Monolith X instrument (NanoTemper Technologies) using premium-treated capillaries at 25 °C. The binding data were analyzed using Monolith software and replotted in Prism.

### Negative stain

Samples were taken from filaments EMSA (with 1 µM RAD54B), followed by 2 times dilution before being applied to glow-discharged 300-mesh carbon-coated copper grids (Agar Scientific), blotted, and stained with 2% uranyl acetate. For each sample, ~200 micrographs were collected on a T12 microscope (FEI) at a magnification of 30,000 using a Rio CMOS camera (Gatan^TM^). Then, micrographs were processed in cryoSPARC for 2D classification.

### Cryo-EM sample preparation

RAD51 (2 µM) was incubated with ssDNA (BA716; 6 µMnt) or dsDNA (BA503-504; 6 µMbp) in filament EMSA buffer (MgCl₂ replaced with CaCl₂ where indicated) at 25 °C for 10 min to form filaments. Filament samples (4 µL) were applied to plasma-cleaned 300-mesh lacey carbon grids (LC300-Au-UL, EM Resolutions). Next, 3 µL of filament sample was replaced with 3 µL of RAD54B-N or RAD54-N (10 µM). Grids were then blotted and plunged into liquid ethane using a Vitrobot Mark IV (FEI) with a blotting force of −2, a waiting time of 90 s, and a blotting time of 2 s.

### Cryo-EM data processing

For all datasets, movies were corrected with MotionCor2 as implemented in RELION5^56^. All subsequent processing was performed in cryoSPARC versions 4.0–4.6^57^.

#### RAD54B-N bound to RAD51–ssDNA filaments (Ca^2+^)

A total of 6082 micrographs were imported into cryoSPARC, and CTF parameters were estimated using PatchCTF. Approximately 5 million particles were initially picked using a combination of template-free filament tracing and template picker, with 2D class averages of RAD51 filaments from a previous screening dataset as templates. Particles were extracted with a box size of 320 × 320 pixels and fourfold-binned for initial processing. Following 2D classification, ~1 million particles were re-extracted with the same box size and two-fold binning. These particles were subjected to 3D reconstruction with template-free helical refinement, using an estimated helical twist of 56° and rise of 16 Å. Refinement optimized these parameters to 55.9° and 16.2 Å. Because the RAD54B N-terminal peptide occupies an equivalent binding site on each RAD51 protomer, it follows the helical symmetry of the filament and was therefore preserved during helical refinement. Duplicate particles were removed, and the remaining set was re-extracted at the same box size without binning. In total, 755,548 particles and the 3D volume from helical refinement were used in non-uniform refinement, yielding a 2.6 Å reconstruction. Map interpretability was further improved with EMReady^58^.

#### RAD54B-N bound to RAD51–ssDNA filaments (Mg^2+^)

A total of 29,547 micrographs were imported into cryoSPARC, and CTF parameters were estimated with PatchCTF. Approximately 1.6 million particles were picked using a combination of template-free filament tracing and template picker, with 2D averages of RAD51 filaments as templates.

For reconstruction of the RAD54B peptide-bound filament structure, particles were extracted with a box size of 360 × 360 pixels and two-fold binned. Following homogeneous refinement, duplicated particles were removed and the remaining particles re-extracted without binning. Approximately 1.2 million particles, together with the reconstruction from helical refinement, were subjected to non-uniform refinement, yielding a 2.9 Å reconstruction. As the RAD54B N-terminal peptide occupies an equivalent binding site on each RAD51 protomer, it follows the helical symmetry of the filament and was preserved during helical refinement.

To resolve the β-domain, particles were extracted with the same box size and fourfold-binned for initial processing. Template-free helical refinement (initial twist 56°, rise 16 Å) converged to a twist of 55.5° and a rise of 16.3 Å, and the resulting map was used as the reference for homogeneous refinement without imposing symmetry. Because the β-domain density is weak, probably due to conformational flexibility and weak interactions, three rounds of focused 3D classification without symmetry were performed using a loose mask encompassing the groove (Supplementary Fig. 2B, 2nd Workflow Path). Owing to the initially weak β-domain density, the first mask was generated by lowering the map threshold in ChimeraX to enhance the globular density in the groove. Density outside this region was removed manually, and the resulting volume was imported into cryoSPARC to generate a soft mask (Supplementary Fig. 2B, 2nd Workflow Path, blue mesh). Following each round of 3D classification, only classes displaying well-defined β-domain density (Supplementary Fig. 2B, 2nd Workflow Path, red) were retained for homogeneous or non-uniform refinement. The refined reconstruction from each round was subsequently used to generate an improved mask for the next round of focused classification, with only particles belonging to classes with well-defined globular density retained. This iterative strategy progressively enriched particles containing well-resolved β-domain density. A final subset of 148,663 particles was re-extracted without binning and refined by non-uniform refinement, yielding a 3.0 Å reconstruction (Supplementary Fig. 2B 2nd Workflow Path). Although the particles are all oriented along the helical axis, the final particles showed a good angular distribution in the plane orthogonal to the helical axis. Map interpretability for both reconstructions was further improved using EMReady^58^.

#### RAD54B-N bound to RAD51–dsDNA filaments (Ca^2+^)

A total of 14,860 micrographs were imported into cryoSPARC, and CTF parameters were estimated with PatchCTF. Approximately 2.3 million particles were picked and extracted with a 360 × 360-pixel box size and fourfold-binned for initial 2D classification. After classification, particles were re-extracted at two-fold binning, split into two subsets, and subjected to template-free helical refinement (initial twist 56°, rise 16 Å). The resulting reconstructions were used as references for homogeneous refinement. As the RAD54B N-terminal peptide occupies an equivalent binding site on each RAD51 protomer, it follows the helical symmetry of the filament and was therefore preserved during helical refinement. To improve the reconstruction of additional density adjacent to the filament groove, two rounds of focused 3D classification were performed using a loose mask encompassing this region, thereby enriching particles containing well-defined RAD54B density. The same iterative mask-generation and focused classification described for the RAD54B–RAD51–ssDNA (Mg^2+^) dataset was applied (Supplementary Fig. 4). The selected particles from both subsets were then combined, re-extracted without binning, subjected to a further round of 3D classification and homogeneous refinement, yielding a final reconstruction at 2.4 Å resolution. Map interpretability was improved with EMReady^58^.

#### RAD54-N bound to RAD51–ssDNA filaments (Ca^2+^)

A total of 8876 micrographs were imported into cryoSPARC, and CTF parameters were estimated using PatchCTF. Approximately 6 million particles were picked using template-free filament tracing, extracted with a box size of 440 × 110 pixels, and fourfold-binned for initial processing. Following 2D classification, ~1.7 million particles were re-extracted without binning and subjected to template-free helical refinement using an initial helical twist of 56° and rise of 16 Å, which converged to 56° and 15.6 Å, respectively. Because the RAD54 N-terminal peptide occupies an equivalent binding site on each RAD51 protomer, it follows the helical symmetry of the filament and was therefore preserved during helical refinement. Duplicate particles were removed, and the remaining 1,388,876 particles together with the refined volume were subjected to a second round of helical refinement, yielding a 3.2 Å reconstruction. Map interpretability was improved with EMReady^58^.

The helical pitch for each final reconstruction was calculated from the optimized helical symmetry parameters obtained by symmetry search in CryoSPARC.

### Model building

Structural models comprising six RAD51 protomers bound to ssDNA or dsDNA, ATP, Ca²⁺ or Mg²⁺, and RAD54B-N or RAD54-N (as appropriate) were generated using AlphaFold^59^. These models were rigid-body docked into the cryo-EM maps, manually adjusted in Coot^60^, and subsequently refined in Phenix^61^.

### Cell biology experiments

#### Cell lines and cell culture

U2OS WT and RAD54B-KO cells were cultured at 37 °C with 5% CO_2_ in Dulbecco’s Modified Eagle’s Medium (DMEM, Sigma) supplemented with 10% fetal calf serum (FCS), 1% Penicillin/Streptomycin, and 1% non-essential amino acids.

#### U2OS RAD54B-KO generation using CRISPR/Cas9

U2OS cells were transfected with pSP-Cas9(BB)−2A-Puro (PX459) V2.0 carrying the sgRNA 5′-CATCTAATATGTATAGGAGC-3′. 24 h later, the medium was changed to selection medium containing 1 µg/ml Puromycin. After nine days, single cells were seeded to obtain colonies. Genomic DNA was isolated using the DNeasy Blood and Tissue Kit (Qiagen). The target region was amplified using Q5 Polymerase and the following primers: fwd 5′-ACTTACTTGCCTCACTGGACTC-3′ and rev 5′-TAAGGTGCCAAAGGGGATT-GG-3′. The knockout was verified by sequencing. For allele 1, sequencing revealed a 2 bp deletion, and therefore, a frameshift starting at aa581, resulting in a premature stop codon at aa588. Allele 2 showed an insertion of 171 bp at aa582, resulting in a premature stop codon after 15 of the inserted base pairs. pSp-Cas9(BB)−2A-Puro (PX459) V2.0 was a gift from Feng Zhang (Addgene plasmid # 62988; http://n2t.net/addgene:62988; RRID: Addgene_62988).

#### Site-directed mutagenesis

A vector encoding the human RAD54B sequence C-terminally tagged with YFP was purchased from Vectorbuilder and subjected to site-directed mutagenic PCR (primer sequences to introduce the different mutations are listed in the table). Three different mutations were induced: a RAD54B 4 A mutant carries four subsequent mutations: K135A, K136A, K137A, and H138A. A RAD54B Δ10 mutant contains a deletion of aa2-10, and a RAD54B Δ25 mutant contains a deletion of aa2-25. The plasmid carrying the wild-type RAD54B sequence was amplified by PCR using either the fwd or rev primer of the desired mutation. The reactions were then pooled, realigned, and digested using the DpnI restriction enzyme. After transformation into VB UltraStable E. Coli (Vectorbuilder), clones were validated by colony PCR and whole-plasmid sequencing.

#### Plasmid transfection

U2OS RAD54B-KO cells were transfected with different plasmids (pRP-EYFP, pRP-RAD54B-EYFP, pRP-RAD54B-4A-EYFP, pRP-RAD54B-Δ10-EYFP, and pRP-RAD54B-Δ25-EYFP) using Lipofectamine 2000 (Thermo Fisher Scientific) according to the manufacturer’s instructions. Experiments were performed 48 h after transfection.

#### Immunofluorescence

Cells were cultured on glass coverslips and fixed with 2.5% formaldehyde in PBS for 15 min at room temperature (RT), washed with PBS-Tween (PBS-T), and permeabilized with 0.2% Triton-X 100 in PBS-T/1% FCS for 5 min at 4 °C. Cells were then washed with PBS-T/1% FCS, blocked for 30 min at RT using 5% BSA/PBS-T/1% FCS, and stained with primary antibodies overnight at 4 °C (γH2AX (1:2,000, ab81299, Abcam) and GFP (1:1,000, 11814460001, Roche)), This was followed by washing with PBS-T/1% FCS and staining using the Click-iT EdU Cell Proliferation Kit (Baseclick, Cy5). Afterward, cells were washed with 3% BSA/PBS and incubated with fluorescently labeled secondary antibodies (goat anti-mouse 488 (A11001) and goat anti-rabbit 594 (A11012), both 1:1000, Molecular Probes) for 1 h at RT. Cells were washed with PBS-T, stained with 0.4 µg/ml DAPI (Sigma Aldrich) in PBS for 5 min at RT, and mounted using Vectashield antifade mounting media (Vector Laboratories). Representative images were taken using a Zeiss microscope and the Metafer4 software (MetaSystems) and subsequently processed using the ImageJ software, where brightness and contrast were adjusted for every picture if necessary.

#### Camptothecin treatment and cell cycle-specific DSB repair analysis

U2OS RAD54B-KO cells were treated with 5 nM Camptothecin (CPT) for 24 h. One hour before fixation, 10 µM of the thymidine analog EdU was added to analyze DSB repair in a cell cycle-specific manner. After fixation, DAPI and EdU intensities were scanned and measured using a Zeiss microscope and the Metafer4 software (MetaSystems). The cell cycle distribution of the scanned cell population was plotted for DNA content (measured by DAPI intensity) and EdU incorporation. EdU-negative G1- and G2-phase cells were differentiated based on their DAPI content. S-phase cells incorporated EdU and were excluded from the analysis. EdU-negative G2-phase cells were analyzed for YFP and γH2AX. γH2AX foci were counted manually in 20-40 cells per condition and experiment.

### Reporting summary

Further information on research design is available in the Nature Portfolio Reporting Summary linked to this article.

## Supplementary information

Supplementary Information

Reporting Summary

Transparent Peer Review file

## Source data

Source Data

## Data Availability

Cryo-EM density maps and atomic models of RAD54B-N–RAD51–ssDNA (Ca^2+^), RAD54B-N–RAD51–dsDNA (Ca^2+^), RAD54B-N–RAD51–ssDNA (peptide, Mg^2+^), RAD54B-N–RAD51–ssDNA (β-domain, Mg^2+^), and RAD54-N–RAD51–ssDNA (Ca^2+^) have been deposited at the Electron Microscopy Data Bank (EMDB) and PDB under the following accession codes: EMD-55166 [https://www.ebi.ac.uk/pdbe/entry/emdb/EMD-−55166] and 9SRZ (RAD54B-N–RAD51–ssDNA (Ca2+)), EMD-56179 [https://www.ebi.ac.uk/pdbe/entry/emdb/EMD-−56179] and 9TRM (RAD54B–N–RAD51–dsDNA (Ca2+)), EMD-55189 and 9SSL (RAD54B–RAD51–ssDNA (peptide, Mg2+)), EMD-56178 and 9TRL (RAD54B–RAD51–ssDNA (β-domain, Mg2+)) and EMD-56447 [https://www.ebi.ac.uk/pdbe/entry/emdb/EMD-−56447] and 9TYY (RAD54-N–RAD51–ssDNA (Ca2+)). RAD54B models generated by AlphaFold (alphafoldserver.org) have been deposited at ModelArchive (modelarchive.org), including RAD54B (β-domain)–RAD51–ssDNA with the accession code ma-5qf74 [https://modelarchive.org/doi/10.5452/ma-5qf74] and RAD54B (β-domain)–RAD51–ssDNA with the accession code ma-hifjh [https://modelarchive.org/doi/10.5452/ma-hifjh]. The AlphaFold model of RAD54B monomer has been obtained from Uniprot (Q9Y620, AF-Q9Y620-F1). Source data are provided with this paper.
