# Supplementary material for: Structures and molecular mechanisms of RAD54B in modulating homologous recombination
Source: Nat Commun. 2026 Oct 12;17:11555. doi: 10.1038/s41467-026-78608-4 (PMC13748634; doi:10.1038/s41467-026-78608-4)
Supplement: Supplementary file 1 [file 41467_2026_78608_MOESM1_ESM.pdf]

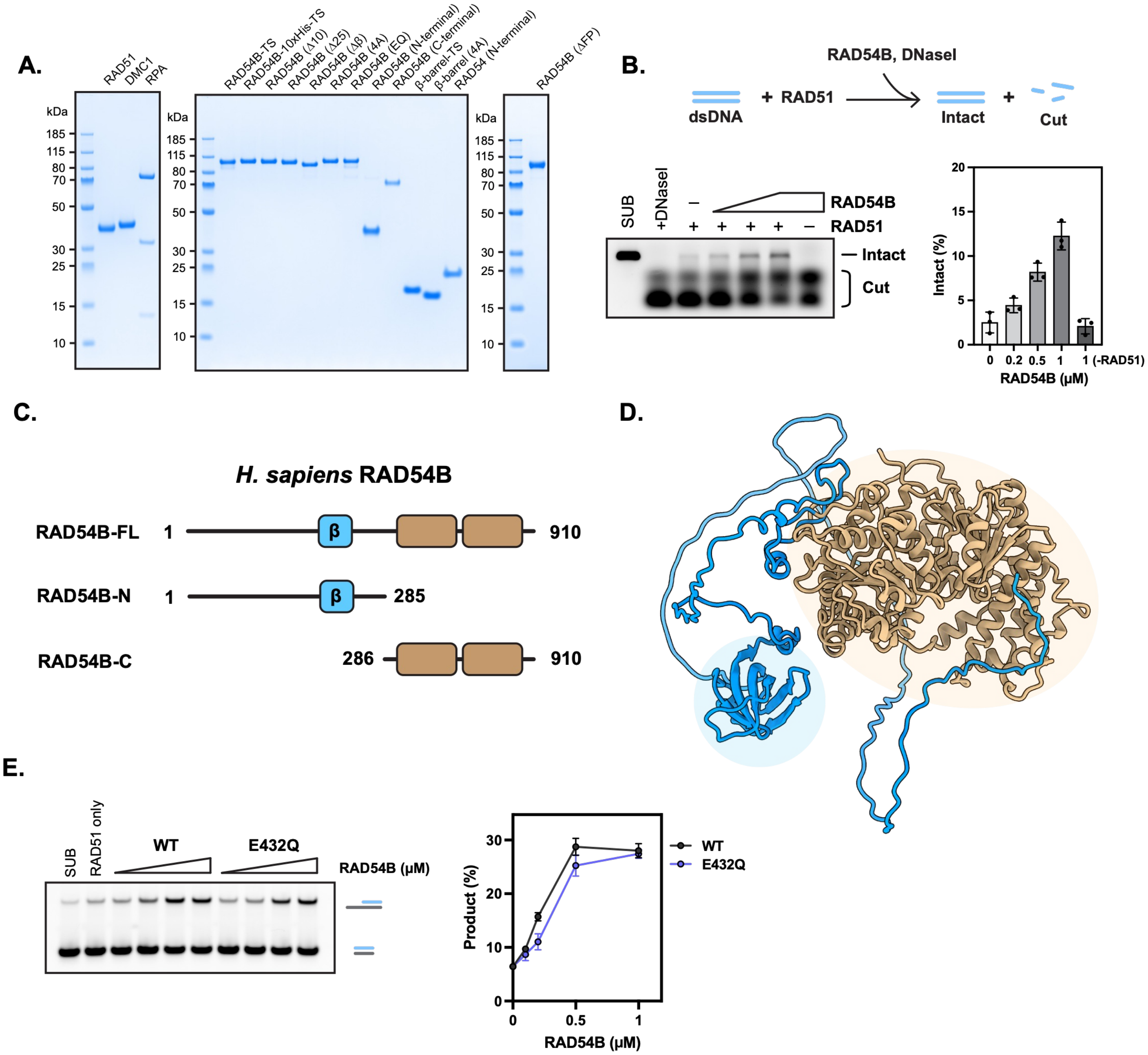

Supplementary Fig. 1. (A) SDS-PAGE analysis of purified proteins in this study. (**B**) nuclease protection assay of RAD51 on dsDNA. **(C)** Domain schematic of RAD54B. **(D)** AlphaFold3 model of human RAD54B. N-terminal region (Blue) and C-terminal region (brown). **(E)** Representative gel images showing the stimulation of RAD51-driven strand exchange by RAD54B (wild-type and mutant), averages are shown with error bars depicting standard deviation (n = 3).

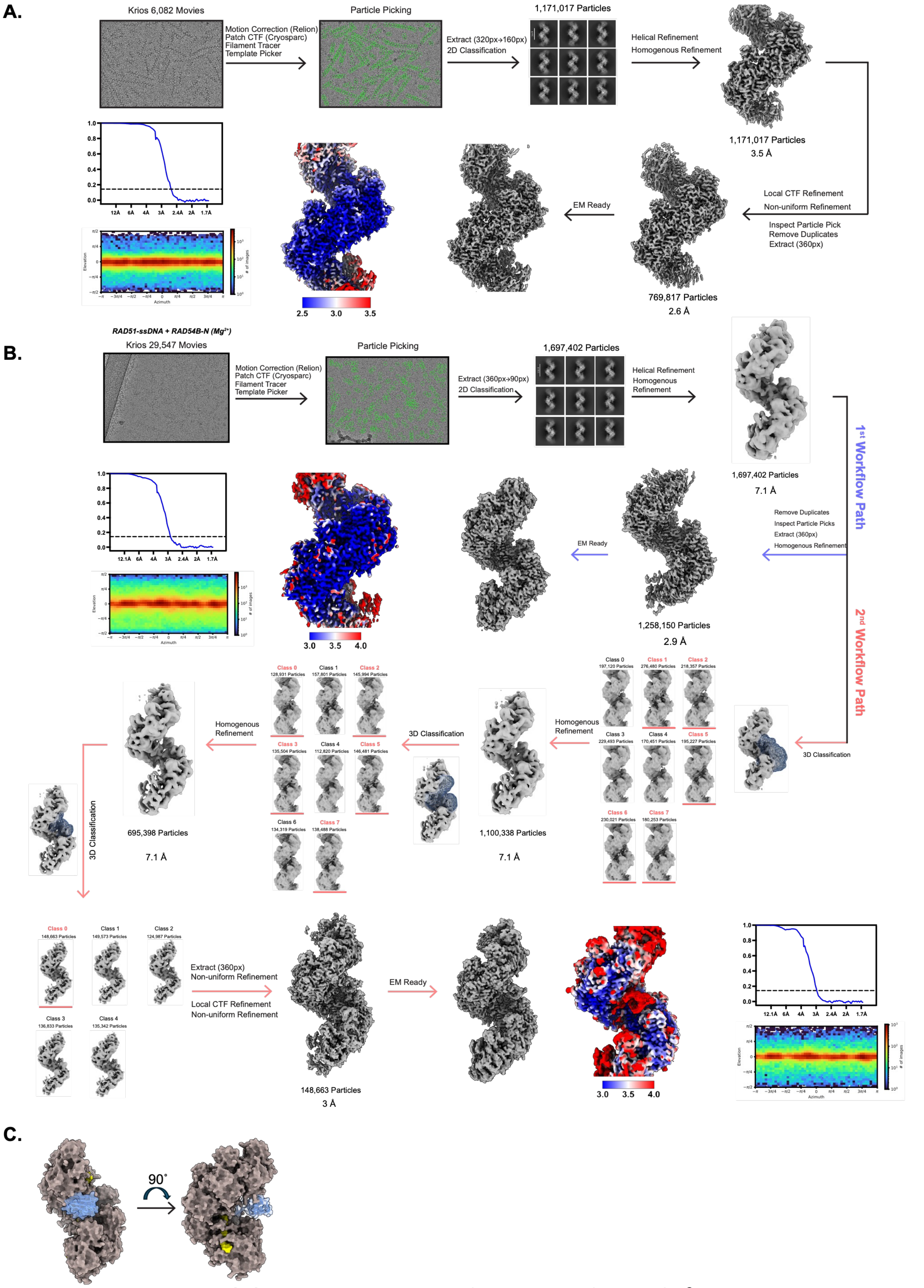

Supplementary Fig. 2. (A) Data processing flowchart of the Ca^{2+}-ATP RAD51-ssDNA filaments bound to RAD54B-N. **(B)** Data processing flowchart of the Mg^{2+}-ATP RAD51-ssDNA filaments bound to RAD54B-N. 1st Workflow pathway (purple) for processing non-β-domain bound structure. 2nd Workflow pathway (salmon) for processing the β-domain bound structure. Masks used in focused classification were show as blue mesh and classes that were retained for subsequent processing were labelled as red. **(C)** well resolved globular density in the RAD51 filament groove. Local resolution maps, FSC curves and particular angular distributions are shown next to the final reconstruction.

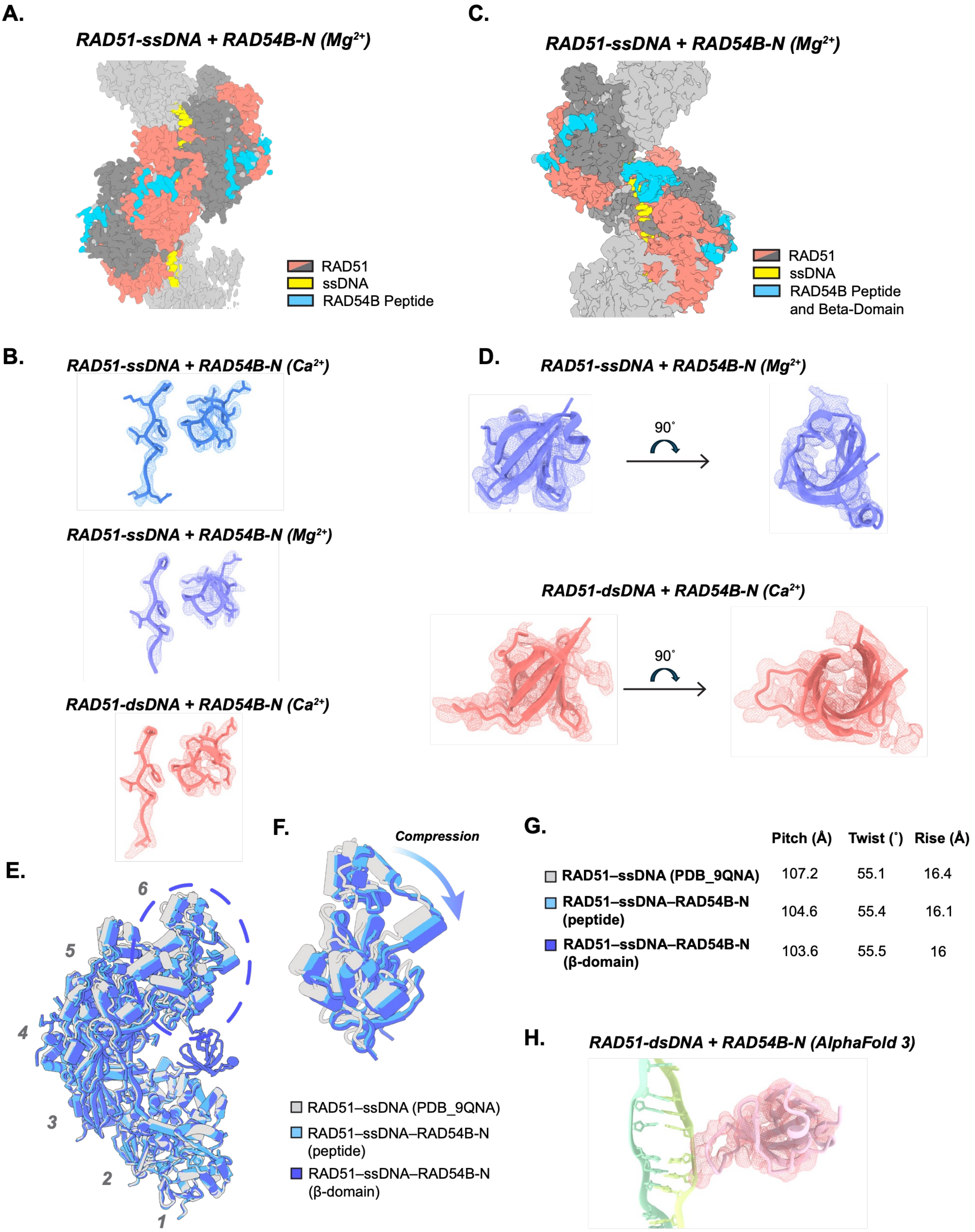

Supplementary Fig. 3. (A) Cryo-EM map of the Mg^{2+}–ATP–bound RAD51-ssDNA filament in complex with RAD54B-N showing peptide density but no β-domain density. (B) Cryo-EM map quality of RAD54B sites 1 and 2 in different complexes from this study. **(C)** Cryo-EM map of the Mg^{2+}–ATP–bound RAD51–ssDNA filament in complex with RAD54B-N showing β-domain density and partial peptide density. (D) Cryo-EM map quality of the RAD54B β-domain in complex with RAD51-ssDNA and RAD51-dsDNA filaments. **(E)** Structural comparison of RAD51-ssDNA filaments with or without RAD54B-N, showing filament compression upon RAD54B-N binding. Three models are aligned to the first RAD51 protomer. (**F**) zoomed in view of (**E**). (**G**) helical parameters of the various filament structures. **(H)** AlphaFold3 model of RAD51-dsDNA filaments with the RAD54B β-domain (RAD51 hidden), showing how the β-domain engages dsDNA and fits the density observed in the cryo-EM map.

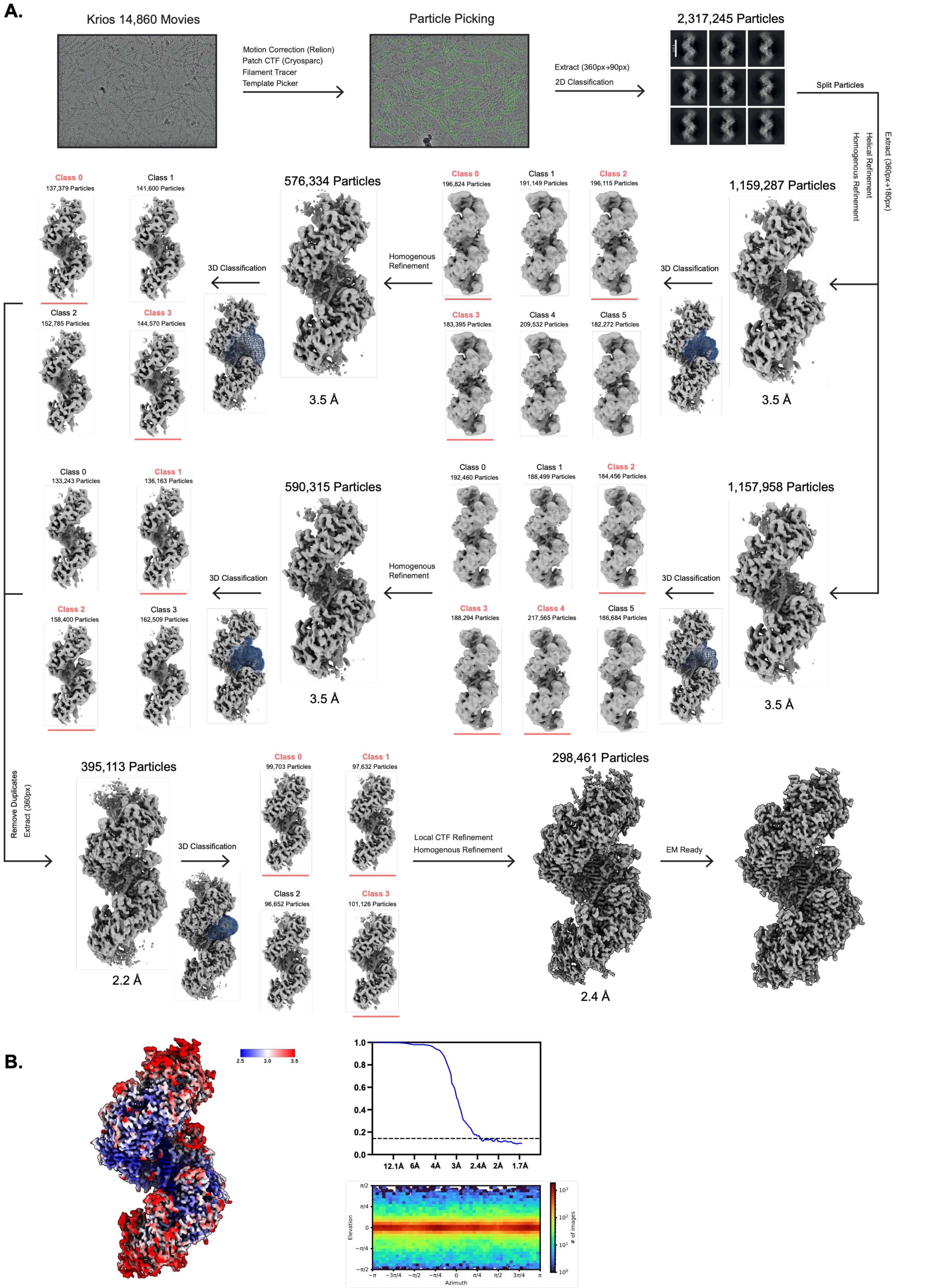

Supplementary Fig. 4 (A) Data processing flowchart of the Ca^{2+}-ATP RAD51-dsDNA filaments bound to RAD54B-N. **(B)** Cryo-EM map of the filament from **(a)** coloured by local resolution. Orientation distribution and FSC curve of the filament from **(A)**. Masks used in focused classification were show as blue mesh and classes that were retained for subsequent processing were labelled as red.

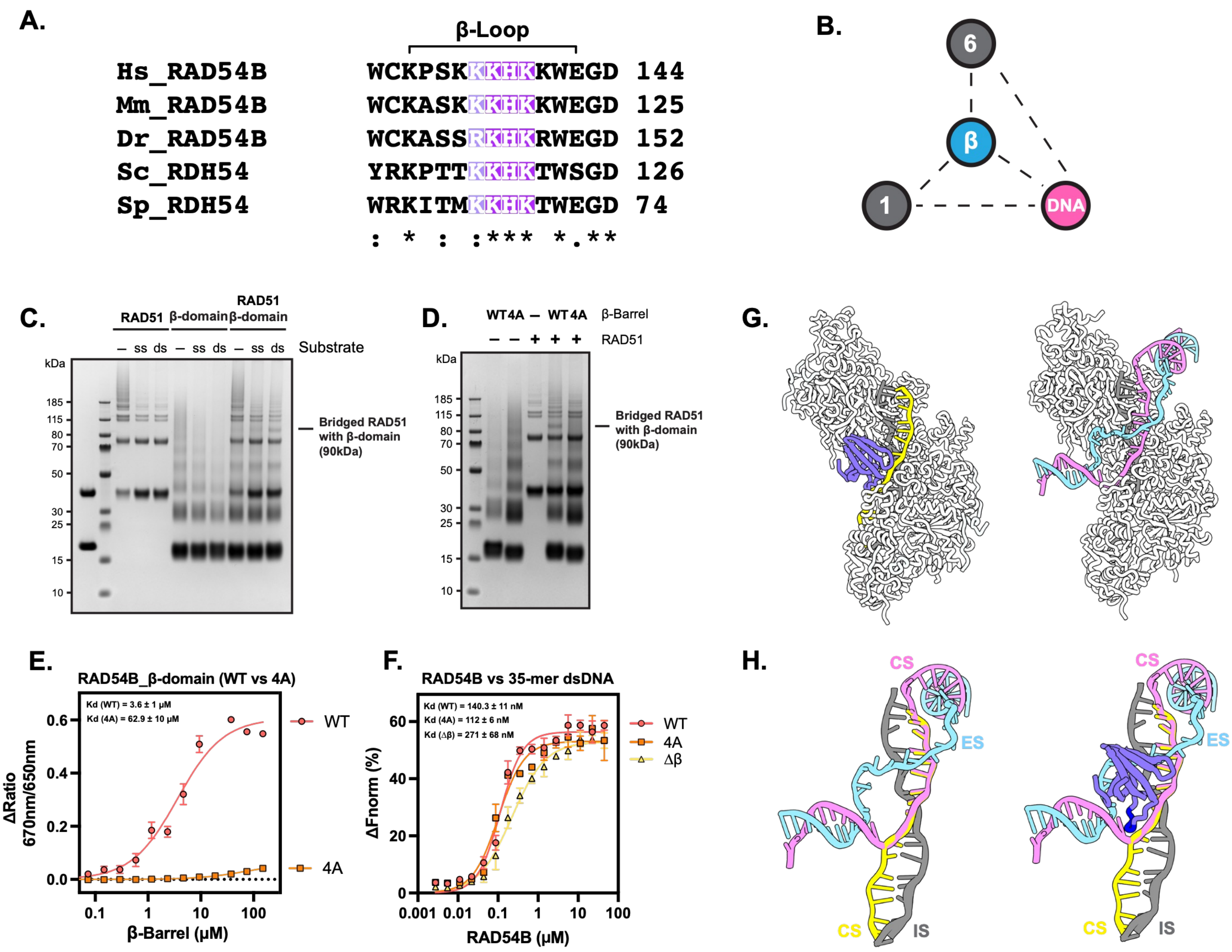

Supplementary Fig. 5 (A) Sequence alignment of the β-loop among RAD54B orthologs. **(B)** Schematic model of the β-domain forming a triangular interaction with two RAD51 protomers (#1 and #6) and dsDNA. **(C)** Full-size gel image corresponding to Fig. 5B. **(D)** Full-size gel image corresponding to Fig. 5D. **(E)** Microscale thermophoresis (MST) binding curves comparing wild-type RAD54B β-domain and the 4A mutant. Spectral shift curves were generated using Monolith software. **(F)** MST binding curves of full-length RAD54B (wild-type and mutants) with 35-mer dsDNA. For **(C-F)**, averages are shown with error bars depicting standard deviation (n = 3). **(G)** Structure of the RAD54B-RAD51-dsDNA filament (left) and the RAD51-D-loop complex (right; PDB: 9SW0). **(H)** Superposition of the two structures in **(G)**, aligned on RAD51, showing that the RAD54B β-domain is positioned to engage the complementary strand during strand invasion. Gray (incoming strand; IS), yellow/pink (complementary strand; CS), blue (exchanged strand; ES).

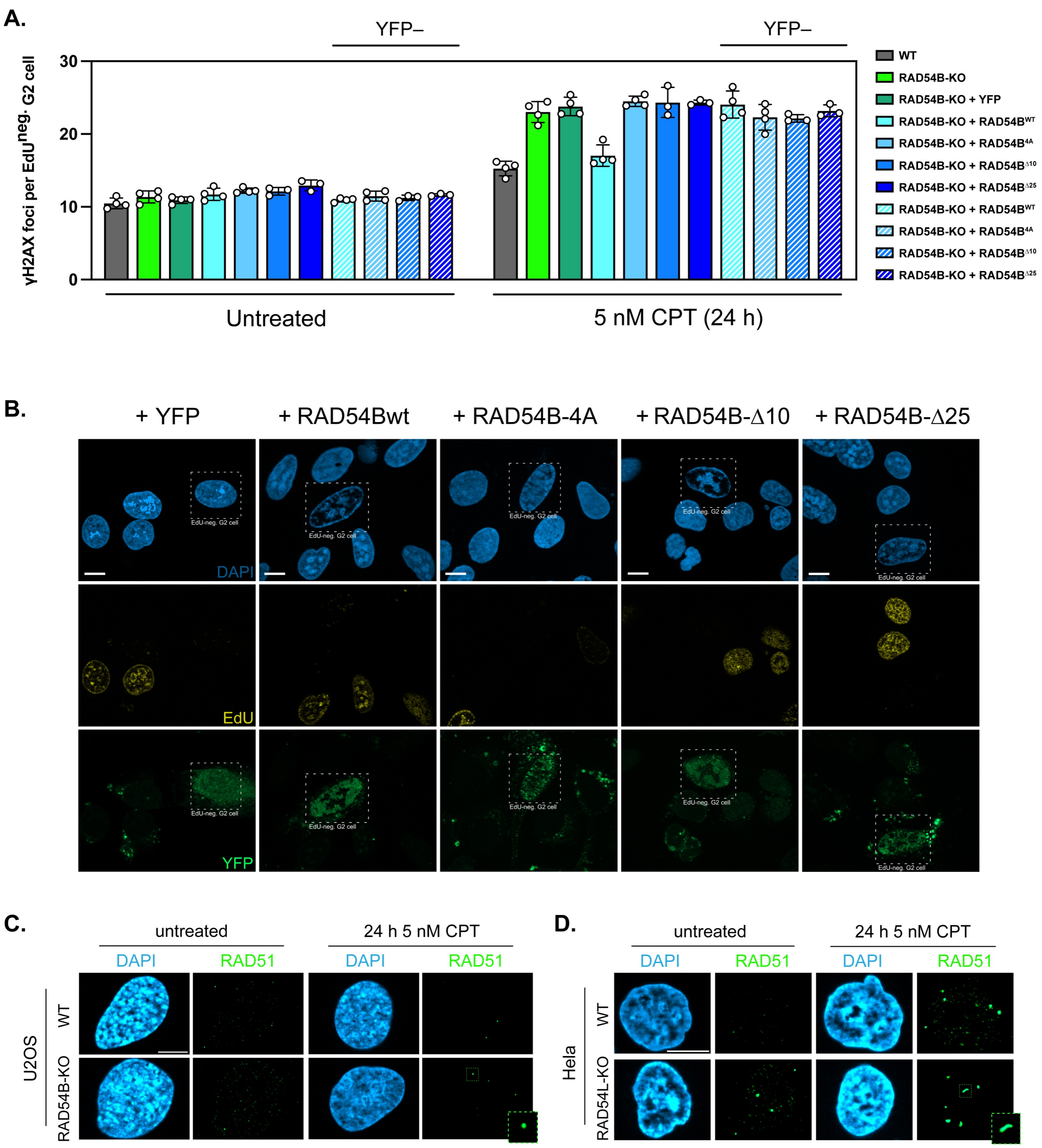

Supplementary Fig. 6 (A) The RAD51 interaction sites and the β-domain of RAD54B are important for DSB repair by HR in U2OS cells. γH2AX foci levels in U2OS WT and RAD54B-KO cells with and without treatment with 5 nM Camptothecin (CPT) for 24 h. RAD54B-KO cells were left untransfected or were transiently transfected with constructs for YFP-only, YFP-tagged WT RAD54B, RAD54B 4A, RAD54B Δ10 or RAD54B Δ25. 1 h before fixation, 10 μM EdU was added to monitor the cell cycle. γH2AX foci were quantified in EdU-negative G2-phase cells. In transfected cells, γH2AX foci were enumerated in YFP-positive (solid bars) and YFP-negative cells (striped bars). Bars represent the mean ± SEM (n=3-4), dots represent single experiments. **(B)** Representative immunofluorescence images of the experiment shown in panel A. Highlighted in each image is a YFP-positive EdU-negative G2-phase cell. The expression of the YFP-tagged WT RAD54B as well as the three mutated versions were similar. The detection of the YFP-signal visualizes a nuclear expression. In contrast, the expression of the YFP-only plasmid was detected in both the nucleus and the cytoplasm (as shown in the image as green signal outside the box). **(C, D)** Representative immunofluorescence images of U2OS WT and RAD54B-KO cells (**C**) and Hela WT and RAD54L-KO cells (**D**). Cells were treated as in panel A and stained for RAD51, EdU and DAPI. Images outlined in green show magnified views of individual RAD51 foci. While RAD51 foci in U2OS WT and RAD54B KO cells have a "normal" round structure, RAD51 foci in HeLa RAD54L KO cells have a "worm-like" RAD51 filament structure. Note that we used U2OS for RAD54B KO cells since these cells depend on RAD54B for repair while HeLa RAD54L KO cells were used because RAD54L is important in this cell type (Chan et al., 2026). Scalebars represent 10 μM.

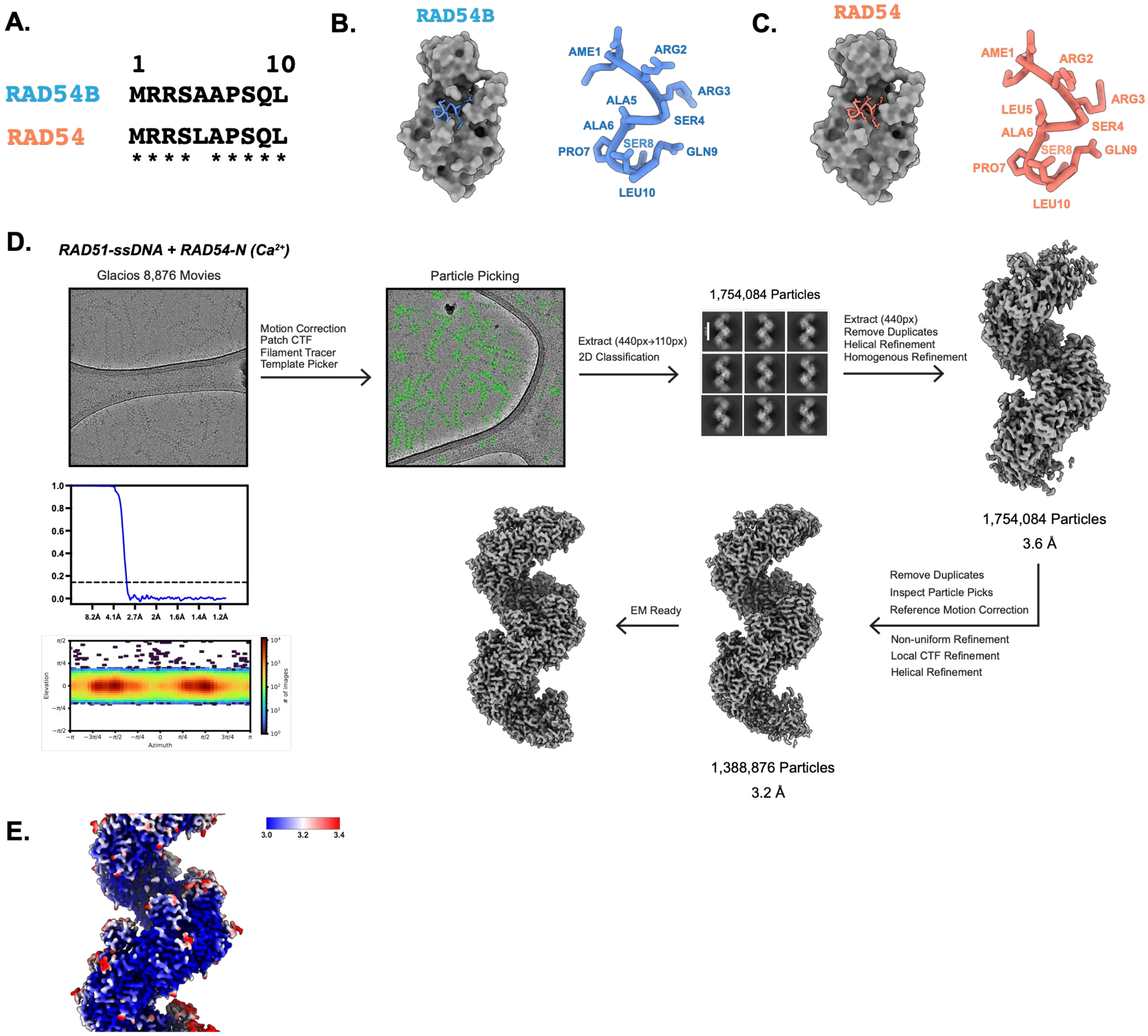

Supplementary Fig. 7 RAD54B share common RAD51 binding sites with other RAD51 modulators. (A) Sequence alignment of the first 10 amino acids of human RAD54 and RAD54B. **(B)** Structural model of RAD54B N-terminal residues (1–10) interacting with a RAD51 protomer. **(C)** Structural model of RAD54 N-terminal residues (1–10) interacting with a RAD51 protomer. **(D)** Data processing flowchart for the Ca^{2+}–ATP RAD51-ssDNA filaments bound to RAD54-N. **(E)** Cryo-EM map of the filament from **(D)**, coloured by local resolution.

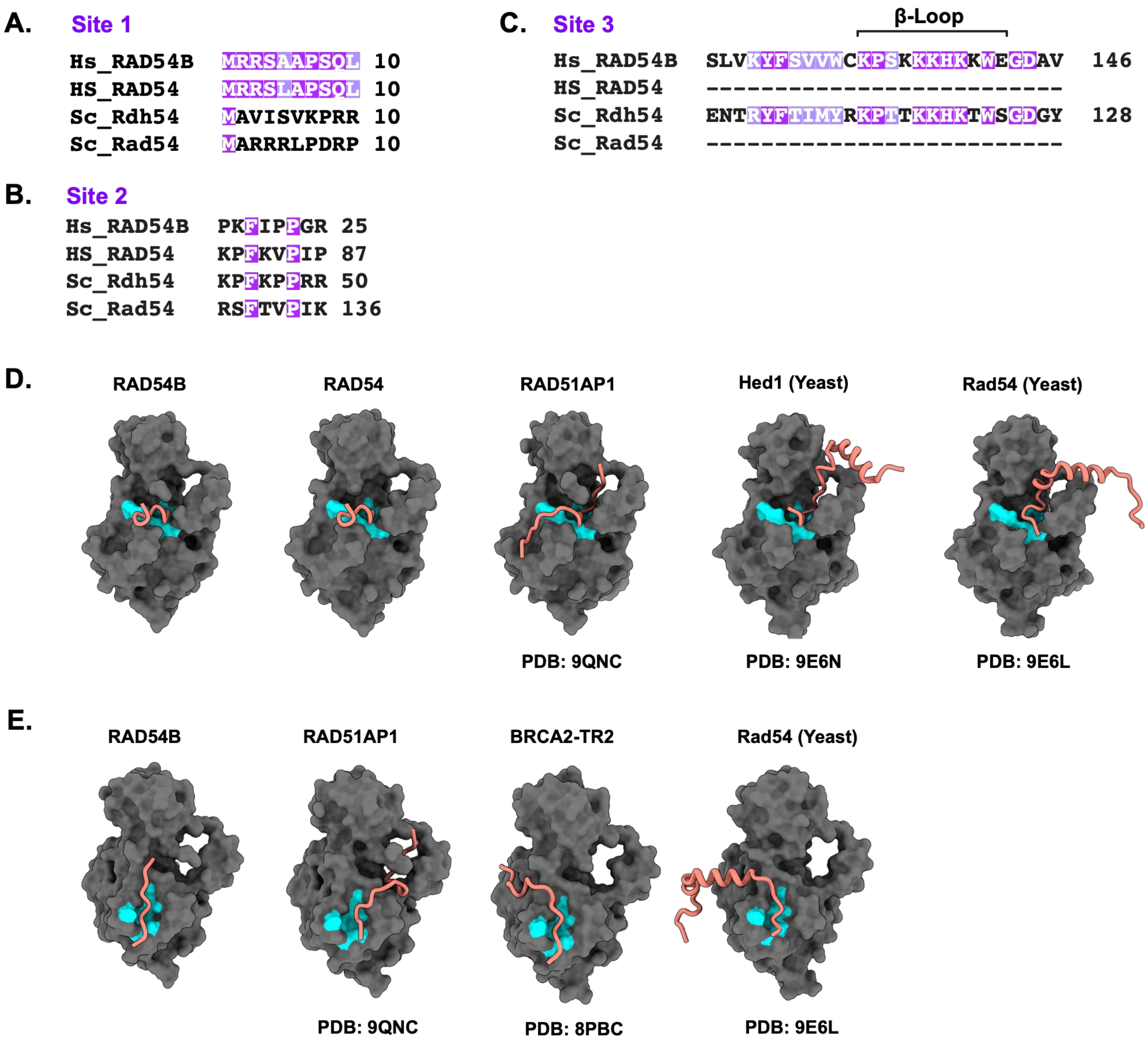

Supplementary Fig. 8 Comparisons of RAD51 binding sites of RAD54B, RAD54 and yeast Rad54 and Rdh54. (A-C) Sequence alignment of the site 1, Site 2 and Site 3 **(D)** Structural comparations of RAD51 modulators known to bind to site 1 (**E**) structural comparisons of known RAD51 modulators having conserved site 2 binding sites.
