# Supplementary material for: Structures and molecular mechanisms of RAD54B in modulating homologous recombination
Source: Nat Commun. 2026 Oct 12;17:11555. doi: 10.1038/s41467-026-78608-4 (PMC13748634; doi:10.1038/s41467-026-78608-4)
Supplement: Supplementary file 2 [file 41467_2026_78608_MOESM2_ESM.pdf]

nature portfolio

Corresponding author(s): Xiaodong Zhang

Last updated by author(s): Sep 2, 2026

Reporting Summary

Nature Portfolio wishes to improve the reproducibility of the work that we publish. This form provides structure for consistency and transparency in reporting. For further information on Nature Portfolio policies, see our Editorial Policies and the Editorial Policy Checklist.

Statistics

For all statistical analyses, confirm that the following items are present in the figure legend, table legend, main text, or Methods section.

n/a	Confirmed	
☐	☒	The exact sample size (*n*) for each experimental group/condition, given as a discrete number and unit of measurement
☐	☒	A statement on whether measurements were taken from distinct samples or whether the same sample was measured repeatedly
☒	☐	The statistical test(s) used AND whether they are one- or two-sided<br>*Only common tests should be described solely by name; describe more complex techniques in the Methods section.*
☒	☐	A description of all covariates tested
☒	☐	A description of any assumptions or corrections, such as tests of normality and adjustment for multiple comparisons
☐	☒	A full description of the statistical parameters including central tendency (e.g. means) or other basic estimates (e.g. regression coefficient) AND variation (e.g. standard deviation) or associated estimates of uncertainty (e.g. confidence intervals)
☒	☐	For null hypothesis testing, the test statistic (e.g. *F*, *t*, *r*) with confidence intervals, effect sizes, degrees of freedom and *P* value noted<br>*Give P values as exact values whenever suitable.*
☒	☐	For Bayesian analysis, information on the choice of priors and Markov chain Monte Carlo settings
☒	☐	For hierarchical and complex designs, identification of the appropriate level for tests and full reporting of outcomes
☒	☐	Estimates of effect sizes (e.g. Cohen's *d*, Pearson's *r*), indicating how they were calculated

Our web collection on statistics for biologists contains articles on many of the points above.

Software and code

Policy information about availability of computer code

Data collection

Cryo-EM data were collected on a Titan Krios or Glacios transmission electron microscope using automated acquisition with EPU (Thermo Fisher Scientific, version 3.11). Detector control and movie acquisition were performed using the manufacturer's software. No open-source or custom code was used for data collection.

Negative stain EM data were collected on a Talos L120C (Twin) using the manufacturer's acquisition software for image recording. No open-source or custom code was used for data collection.

Gel images were acquired using a ChemiDoc Imaging System (Bio-Rad) with the associated acquisition software Image Lab 6.1. No custom or open-source code was used for data collection.

ATPase activity measurements were acquired using a CLARIOstar Plus (BMG Labtech) or SpectraMax iD5 plate reader (Molecular Devices) with the associated acquisition software provided by the manufacturer. No custom or open-source code was used for data collection.

Microscale thermophoresis (MST) data were acquired using a Monolith X instrument (NanoTemper Technologies) with the manufacturer's acquisition software. No custom or open-source code was used for data collection.

Enumeration of gH2AX foci was done in an unbiased fashion with a Zeiss fluorescence microscope, using the semi-automated scanning software Metafer4 (Metasystems v. 3.14.5). To aquire immunofluorescence images, a Zeiss fluorescence microscope with ZEN 3.0 (Blue Edition) software was used. Excel (Microsoft, 365), Graphpad Prism (Dotmatics), Inkscape v1.4.30 and ImageJ (v 1.53c) were used to process the data for publication.

Data analysis	Cryo-EM and negative stain EM data were processed and analysed using cryoSPARC 4.7.1 (Structura Biotechnology). Gel image quantification was performed using Image Lab 6.1 (Bio-Rad), ImageJ 1.54j (NIH), and Microsoft Excel (Microsoft). ATPase activity data were analysed using the manufacturer's software MARS (BMG Labtech) and Microsoft Excel. ATPase assay data for Figure 4D were analysed by Microsoft Excel (Microsoft, 365) and plotted by Graphpad Prism (Dotmatics). Microscale thermophoresis data were analysed using MO.Control 2 (NanoTemper Technologies). No custom code was used for data analysis. gH2AX foci data were analyzed using Metafer4 (Metasystems, v 3.14.5) and processed and visualized using Excel (Microsoft, 365) and Graphpad Prism (Dotmatics).

For manuscripts utilizing custom algorithms or software that are central to the research but not yet described in published literature, software must be made available to editors and reviewers. We strongly encourage code deposition in a community repository (e.g. GitHub). See the Nature Portfolio guidelines for submitting code & software for further information.

Data

Policy information about availability of data

All manuscripts must include a data availability statement. This statement should provide the following information, where applicable:

- Accession codes, unique identifiers, or web links for publicly available datasets
- A description of any restrictions on data availability
- For clinical datasets or third party data, please ensure that the statement adheres to our policy

RAD51-ssDNA filament in complex with calcium and ATP bound by the RAD54B N-terminus (pdb 9SRZ; EMD-55166)
RAD51-ssDNA filament in complex with magnesium and ATP bound by the RAD54B N-terminus (peptide) (PDB 9SSL; EMD-55189)
RAD51-ssDNA filament in complex with magnesium and ATP bound by the RAD54B N-terminus (beta-barrel) (PDB 9TRL; EMD-56178)
RAD51-dsDNA filament in complex with calcium and ATP bound by the RAD54B N-terminus (PDB 9TRM; EMD-56179)
RAD51-ssDNA filament in complex with calcium and ATP bound by the RAD54 N-terminus (PDB 9TYY; EMD-56447)

All original data for Figure 6 A as well as Supplementary figure S5 I are presented as source data.

Research involving human participants, their data, or biological material

Policy information about studies with human participants or human data. See also policy information about sex, gender (identity/presentation), and sexual orientation and race, ethnicity and racism.

Reporting on sex and gender	none
Reporting on race, ethnicity, or other socially relevant groupings	none
Population characteristics	none
Recruitment	none
Ethics oversight	none

Note that full information on the approval of the study protocol must also be provided in the manuscript.

Field-specific reporting

Please select the one below that is the best fit for your research. If you are not sure, read the appropriate sections before making your selection.

☒ Life sciences ☐ Behavioural & social sciences ☐ Ecological, evolutionary & environmental sciences

For a reference copy of the document with all sections, see nature.com/documents/nr-reporting-summary-flat.pdf

Life sciences study design

All studies must disclose on these points even when the disclosure is negative.

Sample size	Sample sizes were chosen based on standard practice in structural biology and biochemical assays, sample availability, and the need to obtain reproducible results across independent experiments. For cryo-EM analysis, the number of particles used for reconstruction was determined empirically during image processing, based on particle quality, classification, map resolution, and convergence of refinement. For biochemical assays, experiments were repeated independently to confirm reproducibility, and representative data or quantified results were included where appropriate. For gH2AX foci analysis, sample size was based on previous experiments to obtain statistical significance and reproducibility. As shown in some of our previous publications (Beucher et al., 2009, Elbakry et al., 2021, Llorens-Agost et al., 2021, Chan et al., 2026), usually 40 cells were analyzed, yielding reliable results. The same logic was followed here, except in some cases, where transfection efficiency was low.
Data exclusions	No data were excluded from the analyses. For cryo-EM data processing, particles were iteratively removed during standard 2D and 3D classification procedures to eliminate poorly aligned or low-quality particles, as part of routine data processing workflows. No additional

	exclusion criteria were applied. For gH2AX foci analysis, no data were excluded from the analyses, except in exceptional cases with low staining quality.
Replication	All experiments were independently repeated to ensure reproducibility, and consistent results were obtained across replicates. Biochemical assays, including ATPase activity measurements, gel-based analyses, gH2AX foci analyses and microscale thermophoresis experiments were performed in independent experiments with comparable outcomes. Cryo-EM analyses yielded consistent structural features following standard image processing and refinement workflows. No findings were identified that could not be reproduced.
Randomization	Randomization was not applicable to this study, as experiments were performed using purified proteins and in vitro biochemical assays rather than distinct experimental groups. Samples were handled consistently across experiments to minimize potential sources of bias.
Blinding	Blinding was not relevant to this study, as experiments were performed using purified proteins in in vitro biochemical assays and structural analyses, without allocation into experimental groups. Data collection and analysis were conducted using standardized and automated procedures where possible. For gH2AX foci analyses, blinding was not relevant as well, as the genotypes or treatments are distinguishable by visible effects.

Reporting for specific materials, systems and methods

We require information from authors about some types of materials, experimental systems and methods used in many studies. Here, indicate whether each material, system or method listed is relevant to your study. If you are not sure if a list item applies to your research, read the appropriate section before selecting a response.

Materials & experimental systems

n/a	Involved in the study
☐	☒ Antibodies
☐	☒ Eukaryotic cell lines
☒	☐ Palaeontology and archaeology
☒	☐ Animals and other organisms
☒	☐ Clinical data
☒	☐ Dual use research of concern
☒	☐ Plants

Methods

n/a	Involved in the study
☒	☐ ChIP-seq
☒	☐ Flow cytometry
☒	☐ MRI-based neuroimaging

Antibodies

Antibodies used	For immunofluorescence:<br>Primary antibodies: GFP (1:1000, 11 814 460 001, Roche), gH2AX (1:2000, 05-636, Merck Millipore).<br>Fluorescently-tagged secondary antibodies: Goat anti mouse AlexaFluor 488 (1:1000, A11001, Molecular Probes), Goat anti rabbit AlexaFluor 594 (1:1000, A11012, Molecular Probes).
Validation	For immunofluorescence:<br>All antibodies used are recommended by the manufacturers for the detection of proteins of human origin. Previous publications from our laboratory have already used these antibodies for the same purpose. See: Juhász et al., 2018, Llorens-Agost et al., 2021.

Eukaryotic cell lines

Policy information about cell lines and Sex and Gender in Research

Cell line source(s)	Insect cell lines (Sf9 and Hi5) were used for recombinant protein expression. Sf9 cells were obtained from Thermo Fisher Scientific, and Hi5 cells were obtained from Expression Systems. Sex is not applicable for these insect-derived cell lines. U2OS WT cells were purchased from DSMZ (Cat# ACC 785) and U2OS RAD54B-KO was established in our laboratory using CRISPR/Cas9.
Authentication	The insect cell lines used in this study (Sf9 and Hi5) were not independently authenticated. U2OS WT cells were authenticated by STR analysis according to the global standard ANSI/ATCC ASN-0002.1-2021 (2021) and resulted in an authentic STR profile of the reference STR database.
Mycoplasma contamination	The insect cell lines used in this study (Sf9 and Hi5) as well as the human cell lines U2OS WT and U2OS RAD54B-KO were tested for mycoplasma contamination and found to be negative.
Commonly misidentified lines<br>(See ICLAC register)	*Name any commonly misidentified cell lines used in the study and provide a rationale for their use.*

Plants

Seed stocks	*Report on the source of all seed stocks or other plant material used. If applicable, state the seed stock centre and catalogue number. If plant specimens were collected from the field, describe the collection location, date and sampling procedures.*
Novel plant genotypes	*Describe the methods by which all novel plant genotypes were produced. This includes those generated by transgenic approaches, gene editing, chemical/radiation-based mutagenesis and hybridization. For transgenic lines, describe the transformation method, the number of independent lines analyzed and the generation upon which experiments were performed. For gene-edited lines, describe the editor used, the endogenous sequence targeted for editing, the targeting guide RNA sequence (if applicable) and how the editor was applied.*
Authentication	*Describe any authentication procedures for each seed stock used or novel genotype generated. Describe any experiments used to assess the effect of a mutation and, where applicable, how potential secondary effects (e.g. second site T-DNA insertions, mosiacism, off-target gene editing) were examined.*
