# Supplementary material for: Structures and molecular mechanisms of RAD54B in modulating homologous recombination
Source: Nat Commun. 2026 Oct 12;17:11555. doi: 10.1038/s41467-026-78608-4 (PMC13748634; doi:10.1038/s41467-026-78608-4)
Supplement: Supplementary file 3 [file 41467_2026_78608_MOESM3_ESM.pdf]

nature portfolio

Peer Review File

Structures and molecular mechanisms of RAD54B in modulating homologous recombination

Corresponding Author: Professor Xiaodong Zhang

This file contains all reviewer reports in order by version, followed by all author rebuttals in order by version.

Version 0:

Reviewer comments:

Reviewer #1

(Remarks to the Author)
In this manuscript, P. Liang et al. investigate the structural basis and molecular mechanisms of RAD54B in homologous recombination (HR). The authors determined atomic-resolution cryo-EM structures of the RAD51 nucleofilament bound to the RAD54B N-terminus with different DNA substrates, as well as 2D class averages of negatively stained RAD51 nucleofilaments in complex with full-length RAD54B. Guided by the structural information, they generated several RAD54B mutants to examine the functional roles of sites I, II, and III in HR. Overall, the results are highly interesting and relevant to the eukaryotic recombination field, and the study provides substantial data that could be suitable for publication.

However, I have several major concerns that should be addressed before the manuscript can be considered for acceptance.

Major Concerns
1. Conclusions and mechanistic interpretations

a.The manuscript primarily focuses on the interaction between the RAD54B N-terminus and the RAD51 nucleofilament. The presented data convincingly support the N-terminal interaction, its functional importance, its role in ATPase inhibition, and requirement for D-loop stabilization. However, I find a discrepancy regarding the proposed role of β-domain in dsDNA engagement and dsDNA capture.
The manuscript states that “it is plausible that this region contributes to donor dsDNA engagement during the homology search.” However, the structural data presented here only demonstrate that the N-terminus interacts with the invading strand of homologous dsDNA in the post-synaptic paired state. There is no direct structural evidence in this study supporting a role in initial dsDNA engagement or dsDNA capture.
Although Figure 5J shows enhanced dsDNA pull-down by the WT protein, this effect could alternatively be explained by stabilization of the D-loop product rather than increased dsDNA capture. Therefore, the authors should either provide additional evidence supporting dsDNA engagement/capture or moderate their conclusions accordingly.

b. The manuscript further proposes that “because β-domain modulates RAD54B ATPase activity and higher-order assembly, it may help regulate the productive association of RAD54B with donor dsDNA, thereby enabling the motor activities required for D-loop formation.” I find this interpretation difficult to follow. In particular, the proposed higher-order assembly appears to be based primarily on cross-linking SDS-PAGE analysis. Even if such assemblies do exist, the mechanism underlying their formation and their functional relevance remain unclear. Moreover, the connection between higher-order assembly and dsDNA association is not sufficiently established by the presented data. The authors should clarify this mechanistic link or temper the interpretation.

c. I also found the comparison between RAD54 and RAD54B confusing and insufficiently developed. The manuscript states: “Interestingly this region is highly conserved in RAD54 (Fig. 6 Extended Data Fig. 1A-E) and we suspected that it would bind to RAD51 in an identical fashion. Indeed the cryoEM structure of the RAD54 N-terminal peptide bound to RAD51 is identical to that of RAD54B and suggests this part of RAD54 might play similar roles (Fig. 6 Extended Ddata Fig. 1B-D)” However, no figure is provided showing the RAD54 structure or sequence conservation in the relevant region. Earlier, the manuscript also states that “RAD54B and RAD54 share conserved C-terminal ATPase domains but N-terminal domains differ.” It is therefore unclear to what extent the N-terminal regions are conserved versus divergent. The authors should more clearly delineate both the similarities and differences between the N-termini of RAD54 and RAD54B. In addition, the Introduction should

explicitly distinguish which previous studies were performed on RAD54 versus RAD54B, and explain to a broader audience the unique features, unresolved questions, and biological significance specific to RAD54B.

d. I also had difficulty understanding Figure 6C-D. RAD54B is approximately three times larger than a RAD51 protomer, whereas the N-terminus alone is roughly comparable in size to RAD51. However, the representation of RAD54B in Figure 6C-D appears disproportionate. Furthermore, the C-terminus, which is approximately twice the size of a RAD51 protomer, should not be directly attached to the RAD51 filament based on the authors' own results. In addition, several mechanistic details depicted in Figure 6C-D (also the RPA part) are not directly supported by experimental evidence presented in this study. The authors should revise the schematic model to better distinguish experimentally supported conclusions from speculative interpretations.

2. Cryo-EM structure determination and reconstruction strategy

The cryo-EM reconstruction strategy requires additional clarification. Helical reconstruction is commonly used for RAD51 nucleofilaments because of their repeating symmetry. However, the RAD54B N-terminus does not appear to follow the helical symmetry of the filament and would therefore be expected to average out during helical processing. The authors should explain in greater detail how masked refinement was implemented to resolve the RAD54B N-terminus and the D-loop-related density. In addition, helical filaments analyzed by single-particle methods typically suffer from strong preferred orientation issues. Recent studies of RAD51–D-loop complexes addressed these limitations by engineering mini-filaments and solving the structures using single-particle analysis. The current manuscript should therefore provide a more detailed methodological explanation and theoretical justification for how the asymmetric regions were successfully reconstructed in this study.

Minor Comment
It is unclear how the authors arrive at the conclusion in line 276: “Overall, these data indicate that RAD54B integrates binding, ATPase regulation, filament architecture, and motor activity through distinct structural elements to control discrete steps of homologous recombination.”

Reviewer #2

(Remarks to the Author)
This manuscript provides a comprehensive and compelling analysis of the interactions between RAD54B and the RAD51 nucleoprotein filament, combining an impressive range of biochemical, structural, and cellular approaches. The cryo-EM data are particularly striking: the visualization of RAD51 filaments in contact with a large protein assembly is remarkably elegant, with clearly defined regions of stable interaction that appear almost ‘illuminated’ . This aspect of the work is not only technically impressive but also highly informative at the mechanistic level. Overall, the study delivers a detailed and convincing characterization of RAD54B function, highlighting its modular organization and its role in regulating RAD51 filaments during homologous recombination. The breadth and coherence of the experimental strategy make the conclusions robust and persuasive. In my view, the manuscript is well suited for publication in Nature Communications after minor revisions, mainly consisting of textual clarifications, small figure adjustments, and additional discussion points that would further strengthen the interpretation of the data.

Results

Figures 1 and 2: D-loop formation and RAD51 activity - The authors state that under their experimental conditions, RAD51 alone does not produce detectable stable D-loops. However, previous studies have reported that RAD51 can generate detectable D-loops under certain conditions. Have you tested these conditions where RAD51 is more active?

Figure 3 / structural interpretation : The statement that RAD54B induces “filament compression” needs clarification to me: it means change in helical pitch, protomer spacing ? How is this quantified?

Figure 4
A: in the pull-down assay, did you also test the RAD54B-ΔFP mutant ? it would be interesting to see if this precise mutation also impacts on the binding to RAD51.
B: the y-axis label is missing.
C-D: RAD51–RAD54B ATPase interplay. The manuscript reports that RAD51 stimulates the ATPase activity of RAD54B (Fig. 4D). However, it is unclear to me how the assay discriminates between RAD51 and RAD54B ATPase activities. In figure 4 C and D, the y axis for ATP assay quantification is either [Pi] (μM) or ATP Hydrolysis (min-1). Why ? could you please clarify ?

Figure 5 : The crosslinking experiment is interesting and supports a bridging role of the β-domain. There is an apparent discrepancy to me: Fig. 5F (EMSA) suggests that the β-domain 4A RAD54 mutant does efficiently bind to dsDNA. But in the manuscript it is written that ’this mutation significantly reduced dsDNA binding by the β-domain (Fig.S5E)’ I don’t see this figS5E and where is this result. ’Extended data Fig. 1E’ is also cited, but I was unable to find it. In this paragraph, there appears to be some confusion in the figure references, and the results are not very easy to follow. Could you please clarify and improve the overall coherence of this section?

Figure 6 : The rationale for restricting the analysis to EdU-negative (presumably G2-phase) cells should be clarified. Given

that camptothecin primarily induces replication-associated double-strand breaks, it is unclear why S-phase (EdU-positive) cells were not included in the analysis.
It is well established that RAD54 depletion or knockout leads to the formation of increased and 'elongated' RAD51 foci or bundle-like RAD51 structures, reflecting defects in RAD51 filament outcome (e.g., Agarwal et al., 2011; Mason et al., 2015). It would be interesting to examine RAD51 foci in RAD54B knockout cells? This analysis could provide important insight into whether RAD54B shares similar regulatory functions in RAD51 filament dynamics.

Discussion
It is well established that RAD54B is a paralog of RAD54 and shares several biochemical activities. There is also evidence suggesting that these proteins may have partially overlapping functions. However, did the authors test if RAD54B possesses an antirecombinase translocase activity similar to that of RAD54, specifically the ability to remove RAD51 from double-stranded DNA in some conditions? In the EMSA experiments, the authors do not observe any destabilizing effect of RAD54B on RAD51–dsDNA filaments. However, if RAD54B were to displace RAD51 by replacing it on the DNA rather than actively dissociating the filament, such an effect might not be detectable in this type of assay. By contrast, in EM analyses, one would expect to observe a decrease in the number or length of RAD51 filaments if such a mechanism were occurring. Have the authors considered this possibility and tested it experimentally? It would therefore be important to discuss this possibility.
The apparent difference between RAD54B and RAD54 in their ability to stabilize or destabilize RAD51 filaments on ssDNA or dsDNA substrates is likely to be functionally significant and merits further discussion, as it could reflect distinct roles in homologous recombination. More generally, the functional redundancy or specialization between RAD54 and RAD54B is not sufficiently discussed to me.

Methods
Crosslinking conditions: 0.1% glutaraldehyde seems high; please justify or reference. For BS3 (bis(sulfosuccinimidyl) suberate), please add a short explanation of what residues it targets and what distances it probes.

Minor typo/general writing
"Extendd Data Fig. 2B" → should be corrected.
Some sections should be written in the past tense rather than present (e.g., protein purification procedure lines 590–595).
Methods/Protein purification : "descried" → "described" ; "Heaprin" → "Heparin"

Reviewer #3

(Remarks to the Author)
In this manuscript, Liang et al. investigate the molecular mechanisms by which RAD54B regulates RAD51-mediated homologous recombination. Using cryo-EM, mutagenesis, biochemical reconstitution, and cellular assays, the authors show that RAD54B binds and stabilizes RAD51-DNA filaments, inhibits RAD51 ATPase activity, and promotes strand invasion, D-loop formation, and strand exchange. They further demonstrate that the RAD54B N-terminal domain is sufficient for RAD51 binding, RAD51 ATPase inhibition, filament stabilization, and strand-exchange stimulation, whereas the C-terminal ATPase/motor domain is required for productive D-loop formation. Structural analyses identify three RAD51-interacting elements within the RAD54B N-terminal region: an extreme N-terminal binding site, a conserved FxPP motif, and a β-domain that bridges distal RAD51 protomers and contacts the DNA backbone in the RAD51-dsDNA filament structure. Through deletion and point-mutant analyses, the authors assign distinct functions to these elements in RAD51 binding, filament stabilization, RAD54B ATPase regulation, dsDNA engagement, D-loop formation, and strand exchange. Finally, they extend these findings to cells by showing that RAD54B knockout U2OS cells exhibit increased γH2AX foci following camptothecin treatment, a phenotype rescued by wild-type RAD54B but not by selected RAD54B mutants.

Altogether, this is a strong, substantial, and potentially important manuscript that provides a compelling structural and biochemical framework for understanding how RAD54B regulates RAD51 filaments and homologous recombination intermediates. The multidisciplinary approach, combining high-resolution cryo-EM structures with mutagenesis, biochemical reconstitution, and cellular validation, is a major strength and yields important mechanistic insight into RAD54B function. With that said, several points would benefit from further clarification, particularly the strength of the cellular HR claims, the distinction between direct structural observations and inferred mechanisms for full-length RAD54B, and the mechanistic interpretation of the β-domain. My specific queries and suggestions are listed below.

1) The proposed model in Fig. 6 is attractive and is generally consistent with the structural and biochemical data, but the mechanistic role of the β-domain would benefit from clarification. Because the cryo-EM structures were obtained with RAD54B-N, whereas productive D-loop formation requires full-length RAD54B and an intact ATPase domain, the authors should more clearly distinguish direct structural observations from inferred mechanisms involving the full-length motor protein. This is particularly important because the β-domain appears to do more than simply capture donor dsDNA. In full-length RAD54B, the 4A mutation and β-domain deletion do not strongly impair bulk dsDNA binding, but they disrupt higher-order RAD54B–dsDNA complexes and substantially reduce dsDNA-dependent ATPase activity. The authors should therefore clarify whether the β-domain promotes D-loop formation primarily by locally engaging donor dsDNA, by organizing higher-order RAD54B–dsDNA assemblies, by stimulating RAD54B motor activity, or by some combination of these mechanisms.

2) The comparison to recent literature could be sharpened. Shin et al. 2025 on Rad54/Hed1 and the Kuhlen et al. RAD51AP1 study from the same group both overlap with the shared RAD51-interface theme. The discussion handles this

reasonably, but the authors could be more direct about what is genuinely new here versus what extends an emerging shared paradigm among RAD51 modulators. In particular, the β-domain appears to be the most novel and RAD54B-specific structural feature, and its distinctiveness relative to RAD54, Hed1, RAD51AP1, BRCA2, and FIGNL1 should be emphasized more clearly.

3) The authors acknowledge that the β-domain is less well resolved in the RAD51-ssDNA filament, yet the manuscript makes fairly specific claims that it bridges distal RAD51 protomers and induces filament compression in this state. This interpretation is plausible, but the evidence appears stronger for the RAD51-dsDNA structure, where the β-domain and β-loop are better resolved and DNA-backbone contacts are more directly supported. The authors should either soften the claims regarding β-domain function in the ssDNA filament or provide quantitative support for the proposed compression, such as changes in helical rise and twist with and without RAD54B-N, ideally comparing the peptide-only and β-domain-bound classes. This would provide a more rigorous basis for the proposed filament-compression mechanism while preserving the overall model.

4) Related to the cellular experiments, the authors should provide clearer validation that the YFP-tagged RAD54B wild-type and mutant proteins are expressed at comparable levels and localize appropriately. This is particularly important because the failure of individual mutants to rescue γH2AX foci could reflect altered expression, stability, localization, or chromatin recruitment rather than a specific mechanistic defect. Given that the cellular rescue assay is used to support the physiological importance of the RAD51-interaction sites and β-domain, this control is important for interpreting the mutant phenotypes.

Minor Comments:
1) The manuscript would benefit from careful editing for grammar, nomenclature, and figure-reference consistency. Examples include “Extendd Data,” “topoisomerasere,” and other typographical issues.

Version 1:

Reviewer comments:

Reviewer #1

(Remarks to the Author)
In this revision, the authors have addressed most of my concerns. However, there are still two aspects that may require further clarification.

First, it is intriguing that the cryo-EM structure of the RAD54B N-terminus bound to RAD51 filament could be resolved, whereas the reported 2D classification showed only helical views with no obvious density corresponding to RAD54B N-terminus. Based on the supplementary figures, this reviewer has no doubt the author did an excellent job in solving cryoEM structure. However, although the authors state that they have “added more details in the data processing,” this reviewer do not find the current description sufficient. Because this dataset represents a key finding of the study, this reviewer believe the data processing workflow should be described in greater detail in the Methods section. Specifically, the authors should clarify how the masks were generated, how 3D classes were selected for subsequent rounds of classification, which ab initio model(s) were used, and whether there was any evidence of preferred orientation in the final reconstruction. In addition, the authors should briefly summarize in the main text the key aspects of the processing strategy that ultimately enabled successful reconstruction (since this is rathe unique to conventional approach), with the detailed workflow provided in the Methods.

Second, the role of RAD54B N-terminus in promoting RAD51 activity requires further clarification. The authors have performed an extensive and well-executed set of biochemical experiments; however, it remains unclear to this reviewer whether the observed enhancement of RAD51 filament stability and consequently RAD51 activity, as stated in several sections of the manuscript is simply a consequence of reduced ATPase activity (i.e., regulation through Site 1), or whether RAD54B N-terminus stabilizes the RAD51 filament through an ATP hydrolysis-independent mechanism, for example by binding to protomers and directly reinforcing filament assembly. Distinguishing between these two possibilities would provide a clearer mechanistic interpretation of the biochemical data and strengthen the conclusions of the study.

Reviewer #2

(Remarks to the Author)
Having carefully reviewed the authors’ responses to the comments and suggestions, I am satisfied that all concerns have been appropriately addressed. The revisions made have further enhanced the quality of the manuscript. Therefore, I recommend acceptance of the article for publication in its present form.

Reviewer #3

(Remarks to the Author)
This revised manuscript from Liang et al. has largely addressed the concerns raised during initial review, particularly with respect to the mechanistic interpretation of the b-domain. I am satisfied with the authors’ revisions and think the manuscript

has improved substantially. I would support publication after minor textual revision addressing the remaining issue below.

My only remaining concern is that some of the β-domain language remains more definitive than the data support. The data support a role for the b-domain in RAD51 filament stabilization, dsDNA interaction in the RAD51-dsDNA structural state, higher-order RAD54B-dsDNA complex formation, RAD54B ATPase regulation, and efficient D-loop formation. However, they do not directly establish the temporal order or causal mechanism by which these activities promote D-loop formation. Donor dsDNA capture and D-loop stabilization should therefore be described as supported mechanistic models, rather than as directly visualized or uniquely demonstrated steps.

This distinction is important because the authors acknowledge in their response that they do not have direct structural evidence that the b-domain promotes initial donor dsDNA capture. Accordingly, statements such as the Abstract phrase that the b-domain ‘contacts donor dsDNA’ should be softened. For example, ‘contacts dsDNA in the RAD51-dsDNA filament structure’ or ‘is positioned to contact donor dsDNA’ would more clearly distinguish the direct structural observation from the inferred mechanism. Similarly, Discussion and Fig. 6 language around dsDNA capture and D-loop stabilization should consistently use wording such as ‘contributes to,’ ‘is positioned to,’ or ‘may stabilize,’ rather than implying that these steps have been directly visualized.

Open Access This Peer Review File is licensed under a Creative Commons Attribution 4.0 International License, which permits use, sharing, adaptation, distribution and reproduction in any medium or format, as long as you give appropriate credit to the original author(s) and the source, provide a link to the Creative Commons license, and indicate if changes were made.
In cases where reviewers are anonymous, credit should be given to 'Anonymous Referee' and the source.
The images or other third party material in this Peer Review File are included in the article’s Creative Commons license, unless indicated otherwise in a credit line to the material. If material is not included in the article’s Creative Commons license and your intended use is not permitted by statutory regulation or exceeds the permitted use, you will need to obtain permission directly from the copyright holder.
To view a copy of this license, visit https://creativecommons.org/licenses/by/4.0/

We thank all the three reviewers for their careful evaluation and expert and insightful critique and suggestions. We have addressed all the concerns they have raised, and we believe the manuscript is improved in terms of data and clarify. We hope that the reviewers are satisfied with our revision and detailed response and the manuscript can now be accepted for publication. Point to point responses are below:

Reviewer #1 (Remarks to the Author):

In this manuscript, P. Liang et al. investigate the structural basis and molecular mechanisms of RAD54B in homologous recombination (HR). The authors determined atomic-resolution cryo-EM structures of the RAD51 nucleofilament bound to the RAD54B N-terminus with different DNA substrates, as well as 2D class averages of negatively stained RAD51 nucleofilaments in complex with full-length RAD54B. Guided by the structural information, they generated several RAD54B mutants to examine the functional roles of sites I, II, and III in HR. Overall, the results are highly interesting and relevant to the eukaryotic recombination field, and the study provides substantial data that could be suitable for publication.
However, I have several major concerns that should be addressed before the manuscript can be considered for acceptance.

Thank you for appreciation of our data and study.

Major Concerns

1. Conclusions and mechanistic interpretations

a. The manuscript primarily focuses on the interaction between the RAD54B N-terminus and the RAD51 nucleofilament. The presented data convincingly support the N-terminal interaction, its functional importance, its role in ATPase inhibition, and requirement for D-loop stabilization. However, I find a discrepancy regarding the proposed role of β-domain in dsDNA engagement and dsDNA capture.
The manuscript states that “it is plausible that this region contributes to donor dsDNA engagement during the homology search.” However, the structural data presented here only demonstrate that the N-terminus interacts with the invading strand of homologous dsDNA in the post-synaptic paired state. There is no direct structural evidence in this study supporting a role in initial dsDNA engagement or dsDNA capture.
Although Figure 5J shows enhanced dsDNA pull-down by the WT protein, this effect could alternatively be explained by stabilization of the D-loop product rather than increased dsDNA capture. Therefore, the authors should either provide additional evidence supporting dsDNA engagement/capture or moderate their conclusions accordingly.

The reviewer is correct that we do not have structural data to support that β-domain promotes dsDNA capture. Figure 5J was conducted using dsDNA that has no homology with ssDNA so there are no D-loop formations so we interpreted the enhanced dsDNA pull-down is due to dsDNA binding by RAD54B when bound to RAD51-ssDNA filaments, consistent with a role in tethering RAD51-ssDNA filaments to the donor dsDNA. Nevertheless, we have modified Discussion substantially.

b. The manuscript further proposes that “because β-domain modulates RAD54B ATPase activity and higher-order assembly, it may help regulate the productive association of RAD54B with donor dsDNA, thereby enabling the motor activities required for D-loop formation.” I find this interpretation difficult to follow. In particular, the proposed higher-order assembly appears to be based primarily on cross-linking SDS-PAGE analysis. Even if such assemblies do exist, the mechanism underlying their formation and their functional relevance remain unclear. Moreover, the connection between higher-order assembly and dsDNA association is not

sufficiently established by the presented data. The authors should clarify this mechanistic link or temper the interpretation.

Thank you for this point and indeed the association between higher order assembly and ATPase activity is weak and require significant more data. We have substantially revised the Discussion (page 16).

c. I also found the comparison between RAD54 and RAD54B confusing and insufficiently developed. The manuscript states: “Interestingly this region is highly conserved in RAD54 (Fig. 6 Extended Data Fig. 1A-E) and we suspected that it would bind to RAD51 in an identical fashion. Indeed the cryoEM structure of the RAD54 N-terminal peptide bound to RAD51 is identical to that of RAD54B and suggests this part of RAD54 might play similar roles (Fig. 6 Extended Ddata Fig. 1B-D)” However, no figure is provided showing the RAD54 structure or sequence conservation in the relevant region. Earlier, the manuscript also states that “RAD54B and RAD54 share conserved C-terminal ATPase domains but N-terminal domains differ.” It is therefore unclear to what extent the N-terminal regions are conserved versus divergent. The authors should more clearly delineate both the similarities and differences between the N-termini of RAD54 and RAD54B. In addition, the Introduction should explicitly distinguish which previous studies were performed on RAD54 versus RAD54B, and explain to a broader audience the unique features, unresolved questions, and biological significance specific to RAD54B.

Thank you. We have modified Introduction to more specifically describe what was known for human, yeast Rad54 and Rad54B. Part of the confusion is the inter-change between yeast and human proteins in the literature and some of the activities were inferred from proteins of one species to another. However, there are clear differences between yeast and human proteins, and between RAD54 and RAD54B. We try to be specific in our description of the literature. We have also substantially modified our Discussion and added a sub-section in Discussion on RAD54 and RAD54B (page 17-18) and included in Supplementary Fig. 8.

d. I also had difficulty understanding Figure 6C-D. RAD54B is approximately three times larger than a RAD51 protomer, whereas the N-terminus alone is roughly comparable in size to RAD51. However, the representation of RAD54B in Figure 6C-D appears disproportionate. Furthermore, the C-terminus, which is approximately twice the size of a RAD51 protomer, should not be directly attached to the RAD51 filament based on the authors’ own results. In addition, several mechanistic details depicted in Figure 6C-D (also the RPA part) are not directly supported by experimental evidence presented in this study. The authors should revise the schematic model to better distinguish experimentally supported conclusions from speculative interpretations.

Thank you and we have modified Fig. 6C.

2. Cryo-EM structure determination and reconstruction strategy

The cryo-EM reconstruction strategy requires additional clarification. Helical reconstruction is commonly used for RAD51 nucleofilaments because of their repeating symmetry. However, the RAD54B N-terminus does not appear to follow the helical symmetry of the filament and would therefore be expected to average out during helical processing. The authors should explain in greater detail how masked refinement was implemented to resolve the RAD54B N-terminus and the D-loop-related density. In addition, helical filaments analyzed by single-particle methods typically suffer from strong preferred orientation issues. Recent studies of

RAD51–D-loop complexes addressed these limitations by engineering mini-filaments and solving the structures using single-particle analysis. The current manuscript should therefore provide a more detailed methodological explanation and theoretical justification for how the asymmetric regions were successfully reconstructed in this study.

We have added more details in data processing.

Minor Comment

It is unclear how the authors arrive at the conclusion in line 276: "Overall, these data indicate that RAD54B integrates binding, ATPase regulation, filament architecture, and motor activity through distinct structural elements to control discrete steps of homologous recombination."

We have revised the manuscript, especially the Discussion substantially.

Reviewer #2 (Remarks to the Author):

This manuscript provides a comprehensive and compelling analysis of the interactions between RAD54B and the RAD51 nucleoprotein filament, combining an impressive range of biochemical, structural, and cellular approaches. The cryo-EM data are particularly striking: the visualization of RAD51 filaments in contact with a large protein assembly is remarkably elegant, with clearly defined regions of stable interaction that appear almost 'illuminated'. This aspect of the work is not only technically impressive but also highly informative at the mechanistic level. Overall, the study delivers a detailed and convincing characterization of RAD54B function, highlighting its modular organization and its role in regulating RAD51 filaments during homologous recombination. The breadth and coherence of the experimental strategy make the conclusions robust and persuasive. In my view, the manuscript is well suited for publication in Nature Communications after minor revisions, mainly consisting of textual clarifications, small figure adjustments, and additional discussion points that would further strengthen the interpretation of the data.

Thank you for the positive evaluation and appreciation of the mechanistic insights this work provides.

Results

Figures 1 and 2: D-loop formation and RAD51 activity - The authors state that under their experimental conditions, RAD51 alone does not produce detectable stable D-loops. However, previous studies have reported that RAD51 can generate detectable D-loops under certain conditions. Have you tested these conditions where RAD51 is more active?

The reviewer is correct that RAD51 alone can generate detectable D-loop product under optimized conditions, as reported previously, and we are able to reproduce similar levels of RAD51 intrinsic activity. However, to investigate the ability of RAD54B to regulate D-loop formation, we optimised conditions so that D-loop formation is sub-optimal with RAD51 alone, while are more sensitive to RAD54B and its various mutants.

Figure 3 / structural interpretation : The statement that RAD54B induces “filament compression” needs clarification to me: it means change in helical pitch, protomer spacing ? How is this quantified?

Yes there are changes in helical pitch and helical twist. We have now included these information in supplementary Fig. 3G and added a superposition of the different structures (Supplementary Fig. 3E-F).

Figure 4

A: in the pull-down assay, did you also test the RAD54B-ΔFP mutant? it would be interesting to see if this precise mutation also impacts on the binding to RAD51.

Yes, we performed ΔFP in the experiment and it does not impact on the binding significantly but together with site 1 deletion, binding is abolished. We have now updated Fig. 4.

B: the y-axis label is missing.

Thank you for pointing this out. We have now included.

C-D: RAD51–RAD54B ATPase interplay. The manuscript reports that RAD51 stimulates the ATPase activity of RAD54B (Fig. 4D). However, it is unclear to me how the assay discriminates between RAD51 and RAD54B ATPase activities. In figure 4 C and D, the y axis for ATP assay quantification is either [Pi] (µM) or ATP Hydrolysis (min-1). Why ? could you please clarify ?

Yes The reviewer is correct that the total ATPase activity is a combination of RAD51 and RAD54B. RAD51 has negligible ATPase activity compared to RAD54B in the presence of dsDNA (Fig. 4E). We have now added a control of RAD54B ATPase deficient mutant (RAD54BKR). Indeed, with this mutant the ATPase activity is at background level (Fig. 4E), supporting that the ATPase activity and stimulation are due to RAD54B. We have now converted Pi to ATPase rate in both Fig. 4D-E.

Figure 5 : The crosslinking experiment is interesting and supports a bridging role of the β-domain. There is an apparent discrepancy to me: Fig. 5F (EMSA) suggests that the β-domain 4A RAD54 mutant does efficiently bind to dsDNA. But in the manuscript it is written that ’this mutation significantly reduced dsDNA binding by the β-domain (Fig.S5E)’ I don’t see this figS5E and where is this result. ’Extended data Fig. 1E’ is also cited, but I was unable to find it. In this paragraph, there appears to be some confusion in the figure references, and the

results are not very easy to follow. Could you please clarify and improve the overall coherence of this section?

We apologise for the clumsy wording. With the full length protein, 4A mutant does not significantly affect dsDNA binding by RAD54B (Supplementary Fig. 5F), probably due to the binding of the other DNA binding domains including the ATPase domain. However, for β-domain alone, which can also weakly bind to dsDNA, 4A mutant shows significant defects (Supplementary Fig. 5E). Figure citations were messed up due to reformatting which we have now corrected.

We have now reworded the sentence on PAGE 12:

"This mutation abolished formation of the 90 kDa crosslinked species (Fig. 5D-E and Supplementary Fig. 5D) and significantly reduced dsDNA binding by the β-domain alone (Supplementary Fig. 5E), supporting a role for the β-loop in connecting distal RAD51 protomers and with dsDNA within RAD51–dsDNA filaments."

Figure 6 : The rationale for restricting the analysis to EdU-negative (presumably G2-phase) cells should be clarified. Given that camptothecin primarily induces replication-associated double-strand breaks, it is unclear why S-phase (EdU-positive) cells were not included in the analysis.

We have compared in the past two different protocols for assessing the repair of replication-associated DSBs. In the first protocol, we treated the cells with a short pulse of CPT and EdU (e.g. 1 h), then removed CPT and EdU and assessed the foci levels after some repair time (e.g. 4-6 h) in EdU-positive cells. In this protocol, cells damaged in early S-phase moved to late S-phase and cells damaged in late S-phase moved to G2-phase during the repair time. In the second protocol, we treated the cells for a longer time with CPT (e.g. overnight), added EdU only 1 h before fixation and analyzed EdU-negative G2-phase cells. In this protocol, all cells in the G2-phase at the time of fixation have moved through the entire S-phase in the presence of CPT and received exactly the same amount of damage and repaired this damage in exactly the same cell cycle phases. We have found the second protocol to provide more consistent data and a lower level of cell-to-cell variability and have therefore applied it in the present study and other projects.

It is well established that RAD54 depletion or knockout leads to the formation of increased and 'elongated' RAD51 foci or bundle-like RAD51 structures, reflecting defects in RAD51 filament outcome (e.g., Agarwal et al., 2011; Mason et al., 2015). It would be interesting to examine RAD51 foci in RAD54B knockout cells? This analysis could provide important insight into whether RAD54B shares similar regulatory functions in RAD51 filament dynamics.

Thank you and we have now performed these experiments. Interestingly, U2OS RAD54B KO cells do not show elongated structures after CPT treatment, while HeLa RAD54 (also called RAD54L) KO cells after the same CPT treatment display elongated RAD51 foci structures, indicating that RAD54B and RAD54L might play key roles in different stages of HR. We have included these in Supplementary Fig. 6 and in text.

Discussion

It is well established that RAD54B is a paralog of RAD54 and shares several biochemical activities. There is also evidence suggesting that these proteins may have partially overlapping functions. However, did the authors test if RAD54B possesses an antirecombinase translocase activity similar to that of RAD54, specifically the ability to remove RAD51 from double-stranded DNA in some conditions? In the EMSA experiments, the authors do not observe any destabilizing effect of RAD54B on RAD51–dsDNA filaments. However, if RAD54B were to displace RAD51 by replacing it on the DNA rather than actively dissociating the filament, such an effect might not be detectable in this type of assay. By contrast, in EM analyses, one would expect to observe a decrease in the number or length of RAD51 filaments if such a mechanism were occurring. Have the authors considered this possibility and tested it experimentally? It would therefore be important to discuss this possibility.

Thank you. We have now performed experiments to directly assess the ability of RAD54B in removing RAD51 from dsDNA and using RAD54 as a control. Contrary to RAD54, RAD54B does not remove RAD51 from dsDNA under these experimental conditions. We have included these data in Fig. 1 (Fig. 1I). Our negative stain EM also shows that the number of RAD51-dsDNA filaments is similar in the absence and presence of RAD54B (Fig. 1D-E). Our results show RAD54B primarily stimulates RAD51 recombinase activities – see Discussion.

The apparent difference between RAD54B and RAD54 in their ability to stabilize or destabilize RAD51 filaments on ssDNA or dsDNA substrates is likely to be functionally significant and merits further discussion, as it could reflect distinct roles in homologous recombination. More generally, the functional redundancy or specialization between RAD54 and RAD54B is not sufficiently discussed to me.

Thank you and we have now extended our Discussion. In addition to modify the Section of comparing binding of RAD51 by different modulators, we have added a Section specifically comparing RAD54, RAD54B and yeast Rad54 and Rdh54 in terms of structures and sequence conservation and potential implications in their distinct functions.

Methods

Crosslinking conditions: 0.1% glutaraldehyde seems high; please justify or reference. For BS3 (bis(sulfosuccinimidyl) suberate), please add a short explanation of what residues it targets and what distances it probes.

The 0.1% glutaraldehyde concentration was selected to stabilize the protein–DNA complexes during sample preparation while avoiding extensive aggregation. This concentration and reaction time were empirically optimized for the complexes used in this study. We have clarified this rationale in the revised Methods and added a reference to glutaraldehyde-based stabilization of protein complexes.

We have included a brief description of the BS3 crosslinker.

Minor typo/general writing

“Extendd Data Fig. 2B” → should be corrected.

Some sections should be written in the past tense rather than present (e.g., protein purification procedure lines 590–595).

Methods/Protein purification : “descried” → “described” ; “Heaprin” → “Heparin”

Have corrected throughout – see track change to the text.

Reviewer #3 (Remarks to the Author):

In this manuscript, Liang et al. investigate the molecular mechanisms by which RAD54B regulates RAD51-mediated homologous recombination. Using cryo-EM, mutagenesis, biochemical reconstitution, and cellular assays, the authors show that RAD54B binds and stabilizes RAD51-DNA filaments, inhibits RAD51 ATPase activity, and promotes strand invasion, D-loop formation, and strand exchange. They further demonstrate that the RAD54B N-terminal domain is sufficient for RAD51 binding, RAD51 ATPase inhibition, filament stabilization, and strand-exchange stimulation, whereas the C-terminal ATPase/motor domain is required for productive D-loop formation. Structural analyses identify three RAD51-interacting elements within the RAD54B N-terminal region: an extreme N-terminal binding site, a conserved FxPP motif, and a β-domain that bridges distal RAD51 protomers and contacts the DNA backbone in the RAD51-dsDNA filament structure. Through deletion and point-mutant analyses, the authors assign distinct functions to these elements in RAD51 binding, filament stabilization, RAD54B ATPase regulation, dsDNA engagement, D-loop formation, and strand exchange. Finally, they extend these findings to cells by showing that RAD54B knockout U2OS cells exhibit increased γH2AX foci following camptothecin treatment, a phenotype rescued by wild-type RAD54B but not by selected RAD54B mutants.

Altogether, this is a strong, substantial, and potentially important manuscript that provides a compelling structural and biochemical framework for understanding how RAD54B regulates RAD51 filaments and homologous recombination intermediates. The multidisciplinary approach, combining high-resolution cryo-EM structures with mutagenesis, biochemical reconstitution, and cellular validation, is a major strength and yields important mechanistic insight into RAD54B function. With that said, several points would benefit from further clarification, particularly the strength of the cellular HR claims, the distinction between direct structural observations and inferred mechanisms for full-length RAD54B, and the mechanistic interpretation of the β-domain. My specific queries and suggestions are listed below.

Thank you for your appreciation of the work, in particularly the multidisciplinary approach and the mechanistic insights provided.

1) The proposed model in Fig. 6 is attractive and is generally consistent with the structural and biochemical data, but the mechanistic role of the β-domain would benefit from clarification. Because the cryo-EM structures were obtained with RAD54B-N, whereas productive D-loop formation requires full-length RAD54B and an intact ATPase domain, the authors should more clearly distinguish direct structural observations from inferred mechanisms involving the full-

length motor protein. This is particularly important because the β-domain appears to do more than simply capture donor dsDNA. In full-length RAD54B, the 4A mutation and β-domain deletion do not strongly impair bulk dsDNA binding, but they disrupt higher-order RAD54B–dsDNA complexes and substantially reduce dsDNA-dependent ATPase activity. The authors should therefore clarify whether the β-domain promotes D-loop formation primarily by locally engaging donor dsDNA, by organizing higher-order RAD54B–dsDNA assemblies, by stimulating RAD54B motor activity, or by some combination of these mechanisms.

We thank the reviewer for the insightful comments and observation. Indeed, we believe β-domain plays multiple roles, including contributing to RAD54B higher order assembly and ATPase activity, as well as capturing dsDNA and interacting with dsDNA and D-loop structure. The observed stimulation in D-loop formation could be due to all the functions: via capturing dsDNA and therefore promoting initial engagement with dsDNA; via ATPase activity, which appears to be important for D-loop formation although the exact mechanism is currently unclear; and via stabilising D-loop as the b-domain could bind to the dsDNA in the D-loop and therefore stabilise the D-loop. We have now incorporated these ideas into the cartoon (Fig. 6C) and in Discussion.

2) The comparison to recent literature could be sharpened. Shin et al. 2025 on Rad54/Hed1 and the Kuhlen et al. RAD51AP1 study from the same group both overlap with the shared RAD51-interface theme. The discussion handles this reasonably, but the authors could be more direct about what is genuinely new here versus what extends an emerging shared paradigm among RAD51 modulators. In particular, the β-domain appears to be the most novel and RAD54B-specific structural feature, and its distinctiveness relative to RAD54, Hed1, RAD51AP1, BRCA2, and FIGNL1 should be emphasized more clearly.

Thank you. We have now included direct comparisons of available structural data and sequence alignments of human RAD54, RAD54B, yeast Rad54 and yeast Rad54B (Supplementary Fig. 8) and expanded our Discussion and potential functional implications.

3) The authors acknowledge that the β-domain is less well resolved in the RAD51-ssDNA filament, yet the manuscript makes fairly specific claims that it bridges distal RAD51 protomers and induces filament compression in this state. This interpretation is plausible, but the evidence appears stronger for the RAD51-dsDNA structure, where the β-domain and β-loop are better resolved and DNA-backbone contacts are more directly supported. The authors should either soften the claims regarding β-domain function in the ssDNA filament or provide quantitative support for the proposed compression, such as changes in helical rise and twist with and without RAD54B-N, ideally comparing the peptide-only and β-domain-bound classes. This would provide a more rigorous basis for the proposed filament-compression mechanism while preserving the overall model.

We have now included figures and helical rise and twist parameters with and without RAD54B-N, separating classes with peptide-only and β-domain-bound classes (Supplementary Fig. 3E-G), showing RAD54B-N binding induces twist and reduces helical pitch (and rise) and the changes are bigger in the class where clear density for β-domain is observed.

4) Related to the cellular experiments, the authors should provide clearer validation that the YFP-tagged RAD54B wild-type and mutant proteins are expressed at comparable levels and localize appropriately. This is particularly important because the failure of individual mutants to rescue γH2AX foci could reflect altered expression, stability, localization, or chromatin recruitment rather than a specific mechanistic defect. Given that the cellular rescue assay is used to support the physiological importance of the RAD51-interaction sites and β-domain, this control is important for interpreting the mutant phenotypes.

Thank for the comments. Indeed, this is something we watched out for. We monitored the YFP levels, which are indicative of expression levels of RAD54B. Wildtype and mutants showed similar levels, suggesting similar levels of proteins were produced. We have now included new images clarifying this aspect (Supplementary Fig. 6B). It is more difficult to monitor if chromatin recruitment was affected but the nuclear distribution and duration of YFP were similar in WT and mutants (Supplementary Fig. 6B), suggesting that the stability and nuclear localisations were not altered significantly. As a control, we have expressed a GFP-only construct which provided a pan-cellular instead of a nuclear signal (Supplementary Fig. 6B).

Minor Comments:

1) The manuscript would benefit from careful editing for grammar, nomenclature, and figure-reference consistency. Examples include “Extendd Data,” “topoisomerasere,” and other typographical issues.

Thank you and this and others have been corrected (see track changes to the text).

We thank the reviewers for their thorough assessment. We have taken aboard their recommendations. Point to point responses are below:

Reviewer #1 (Remarks to the Author):

In this revision, the authors have addressed most of my concerns. However, there are still two aspects that may require further clarification.

First, it is intriguing that the cryo-EM structure of the RAD54B N-terminus bound to RAD51 filament could be resolved, whereas the reported 2D classification showed only helical views with no obvious density corresponding to RAD54B N-terminus. Based on the supplementary figures, this reviewer has no doubt the author did an excellent job in solving cryoEM structure. However, although the authors state that they have "added more details in the data processing," this reviewer do not find the current description sufficient. Because this dataset represents a key finding of the study, this reviewer believe the data processing workflow should be described in greater detail in the Methods section. Specifically, the authors should clarify how the masks were generated, how 3D classes were selected for subsequent rounds of classification, which ab initio model(s) were used, and whether there was any evidence of preferred orientation in the final reconstruction. In addition, the authors should briefly summarize in the main text the key aspects of the processing strategy that ultimately enabled successful reconstruction (since this is rathe unique to conventional approach), with the detailed workflow provided in the Methods.

We have added further details in text and Methods and highlighted the classes that were retained in Supplementary Figs. 2 and 4. There is a good angular distribution in the plane perpendicular to the helical axis as shown in Supplementary Figs. 2 and 4.

Second, the role of RAD54B N-terminus in promoting RAD51 activity requires further clarification. The authors have performed an extensive and well-executed set of biochemical experiments; however, it remains unclear to this reviewer whether the observed enhancement of RAD51 filament stability and consequently RAD51 activity, as stated in several sections of the manuscript is simply a consequence of reduced ATPase activity (i.e., regulation through Site 1), or whether RAD54B N-terminus stabilizes the RAD51 filament through an ATP hydrolysis-independent mechanism, for example by binding to protomers and directly reinforcing filament assembly. Distinguishing between these two possibilities would provide a clearer mechanistic interpretation of the biochemical data and strengthen the conclusions of the study.

We have performed and included date of site 2 deletion (see new Fig. 4) and made the description more specifically (see revised text in track changes). Briefly, site 1 contributes to the inhibition of RAD51 ATPase activity, while also binds to the RAD51 N-terminal domain which is at the protomer-protomer interface, so site 1 probably contributes to filaments stabilisation by both inhibiting ATPase activity and strengthening protomer-protomer interactions. Site 2 also contributes to filament stability but does not appear to contribute to inhibiting RAD51 ATPase activity so it uses an ATPase independent mechanism in stabilising RAD51 filaments. Since it binds at the edge of the filament across the RAD51 ATPase domain, it might contribute to filament stabilisation by preventing filament dissociation. Site 3 (β-

domain) contacts distal protomers so probably contributes to filament stabilisation via these additional interactions. We have made these descriptions clearer.

Reviewer #2 (Remarks to the Author):

Having carefully reviewed the authors' responses to the comments and suggestions, I am satisfied that all concerns have been appropriately addressed. The revisions made have further enhanced the quality of the manuscript. Therefore, I recommend acceptance of the article for publication in its present form.

Thank you.

Reviewer #3 (Remarks to the Author):

This revised manuscript from Liang et al. has largely addressed the concerns raised during initial review, particularly with respect to the mechanistic interpretation of the b-domain. I am satisfied with the authors' revisions and think the manuscript has improved substantially. I would support publication after minor textual revision addressing the remaining issue below.

My only remaining concern is that some of the β-domain language remains more definitive than the data support. The data support a role for the b-domain in RAD51 filament stabilization, dsDNA interaction in the RAD51-dsDNA structural state, higher-order RAD54B-dsDNA complex formation, RAD54B ATPase regulation, and efficient D-loop formation. However, they do not directly establish the temporal order or causal mechanism by which these activities promote D-loop formation. Donor dsDNA capture and D-loop stabilization should therefore be described as supported mechanistic models, rather than as directly visualized or uniquely demonstrated steps.

This distinction is important because the authors acknowledge in their response that they do not have direct structural evidence that the b-domain promotes initial donor dsDNA capture. Accordingly, statements such as the Abstract phrase that the b-domain 'contacts donor dsDNA' should be softened. For example, 'contacts dsDNA in the RAD51-dsDNA filament structure' or 'is positioned to contact donor dsDNA' would more clearly distinguish the direct structural observation from the inferred mechanism. Similarly, Discussion and Fig. 6 language around dsDNA capture and D-loop stabilization should consistently use wording such as 'contributes to,' 'is positioned to,' or 'may stabilize,' rather than implying that these steps have been directly visualized.

We have modified accordingly. See revised text in track changes.
